# Allosteric inhibitor of β-catenin selectively targets oncogenic Wnt signaling in colon cancer

**DOI:** 10.1038/s41598-020-60784-y

**Published:** 2020-05-15

**Authors:** Anton Cheltsov, Natsuko Nomura, Venkata M. Yenugonda, Jatin Roper, Rajesh Mukthavaram, Pengfei Jiang, Nam-Gu Her, Ivan Babic, Santosh Kesari, Elmar Nurmemmedov

**Affiliations:** 1 Q-MOL LLC, San Diego, California, United States of America; 2 John Wayne Cancer Institute and Pacific Neuroscience Institute at Providence Saint John’s Health Center, Santa Monica, CA USA; 3 Division of Gastroenterology, Department of Medicine, Duke University, Durham, North Carolina 27710 USA; 4 Translational Neuro-Oncology Laboratories, Department of Neurosciences, University of California San Diego, La Jolla, CA USA; 5 Korea Institute of Radiological and Medical Sciences, 75 Nowon-ro, Seoul, 01812 Korea

**Keywords:** Small molecules, Virtual screening

## Abstract

Abnormal regulation of β-catenin initiates an oncogenic program that serves as a main driver of many cancers. Albeit challenging, β-catenin is an attractive drug target due to its role in maintenance of cancer stem cells and potential to eliminate cancer relapse. We have identified C2, a novel β-catenin inhibitor, which is a small molecule that binds to a novel allosteric site on the surface of β-catenin. C2 selectively inhibits β-catenin, lowers its cellular load and significantly reduces viability of β-catenin-driven cancer cells. Through direct binding to β-catenin, C2 renders the target inactive that eventually activates proteasome system for its removal. Here we report a novel pharmacologic approach for selective inhibition of β-catenin via targeting a cryptic allosteric modulation site. Our findings may provide a new perspective for therapeutic targeting of β-catenin.

## Introduction

Wnt signaling pathway regulates expression of numerous genes that control development and tissue homeostasis^1–3^. The APC complex tightly regulates the cellular concentration of β-catenin. This is a dynamic assembly that is composed of GSK3β, CK1α, APC and AXIN. Phosphorylation activity of GSK3β and CK1α marks β-catenin at S33, S37 and T41, which in turn signals its recycling through ubiquitin-proteasome pathway^4,5^. Alternative phosphorylation by AKT and PKM1/2 at residues S552, Y654 and S675 may on the other hand enhance the activity of β-catenin^6,7^. Binding of Wnt ligand to Frizzled and LRP5/6 is known to halt the APC complex, leading to translocation of β-catenin into the nucleus. Increased nuclear presence of β-catenin activates the TCF/LEF1-mediated oncogenic events leading to increased stem cell-like behavior in cancers and poor clinical prognosis^8,9^.

Aberrant Wnt signaling has been closely associated to carcinogenesis^10^. Mutations in the pathway components, including APC, Axin, and β-catenin, have been closely linked with various cancers^11–14^. APC plays a pivotal role in β-catenin-dependent tumorigenesis, such that partial or complete loss of its function is recognized as a hallmark of many colon cancers. Impaired APC function leads to over-expression of β-catenin, which in turn renders cancer cells sensitive to its inhibition^15,16^. This vulnerability of many colon cancers is at the core of new therapeutic approaches for β-catenin, and may serve as a source of selectivity for novel β-catenin inhibitors. Indeed inhibition of β-catenin has been in the focus of a number of recent studies^17^. Some of the early Wnt pathway inhibitors are ICG-001, XAV939 and pyrvinium that target CREB protein (CBP), tankyrase and CK1α, respectively. Although these are valuable as research tools, they have limited therapeutic utility^8,17^. Recent efforts have been spent on targeting the β-catenin/TCF4 complex, i.e. the catenin responsive transcription (CRT), with an expectation to inhibit its downstream signaling. The CRT complex interacts with several gene regulators through a conserved mechanism, thus raising questions about specificity of this approach^17^. One recent study reports discovery of inhibitors iCRT-3, -5 and -14 that target the CRT complex^18^. While this provides hope for direct targeting of β-catenin, it is not clear whether they trigger degradation of β-catenin, thus raising concerns about the possibility of its re-localization back into the nucleus. Yet another promising study reports discovery of a small molecule MSAB, which allegedly promotes degradation of β-catenin and slows its oncogenic activity at high concentrations^19^. A more specific inhibitor, stapled peptide StAx35R, is reported to block AXIN from binding to β-catenin^20^. Whereas the peptide is large enough to cover a significant surface area on the target, it carries the drawback of low cell penetration thus necessitating its use at high doses. The clinical advantage of the stapled peptide class of molecules has yet to be established^17^.

While there has been an encouraging increase in the number of Wnt inhibitors, their mechanism of action of these compounds needs to be further elucidated to assess suitability for therapeutic use. As much as Wnt signaling remains attractive for therapeutic intervention, much attention needs to be allocated to therapeutic index and unwanted cell toxicity. It is therefore pharmacologically more meaningful to directly target β-catenin, rather than interfere with its upstream partners that are involved in other essential cellular functions. Direct targeting of β-catenin will eliminate toxicity and increase therapeutic benefit^21^. We report discovery of C2, an experimental small molecule that inhibits β-catenin via direct binding to an allosteric site. C2 reduces the oncogenic pressure of β-catenin and triggers its degradation in β-catenin-overexpressing cancer cells. We anticipate that our findings will contribute to the understanding of basic β-catenin biology, as well as encourage further drug discovery.

## Results

### Discovery and characterization of C2

Focused computational analysis has revealed a cryptic allosteric site, site C, which is not involved in canonical interactions known to drive Wnt signaling pathway (Supplementary Fig. 1). Site C spans across armadillo domains 8 through 10, which is functionally isolated from the binding sites of TCF4, AXIN1 and BCL9. This region is characterized by binding interactions with affinities in upwards of 30 nM^22,23^.

**Figure 1:**
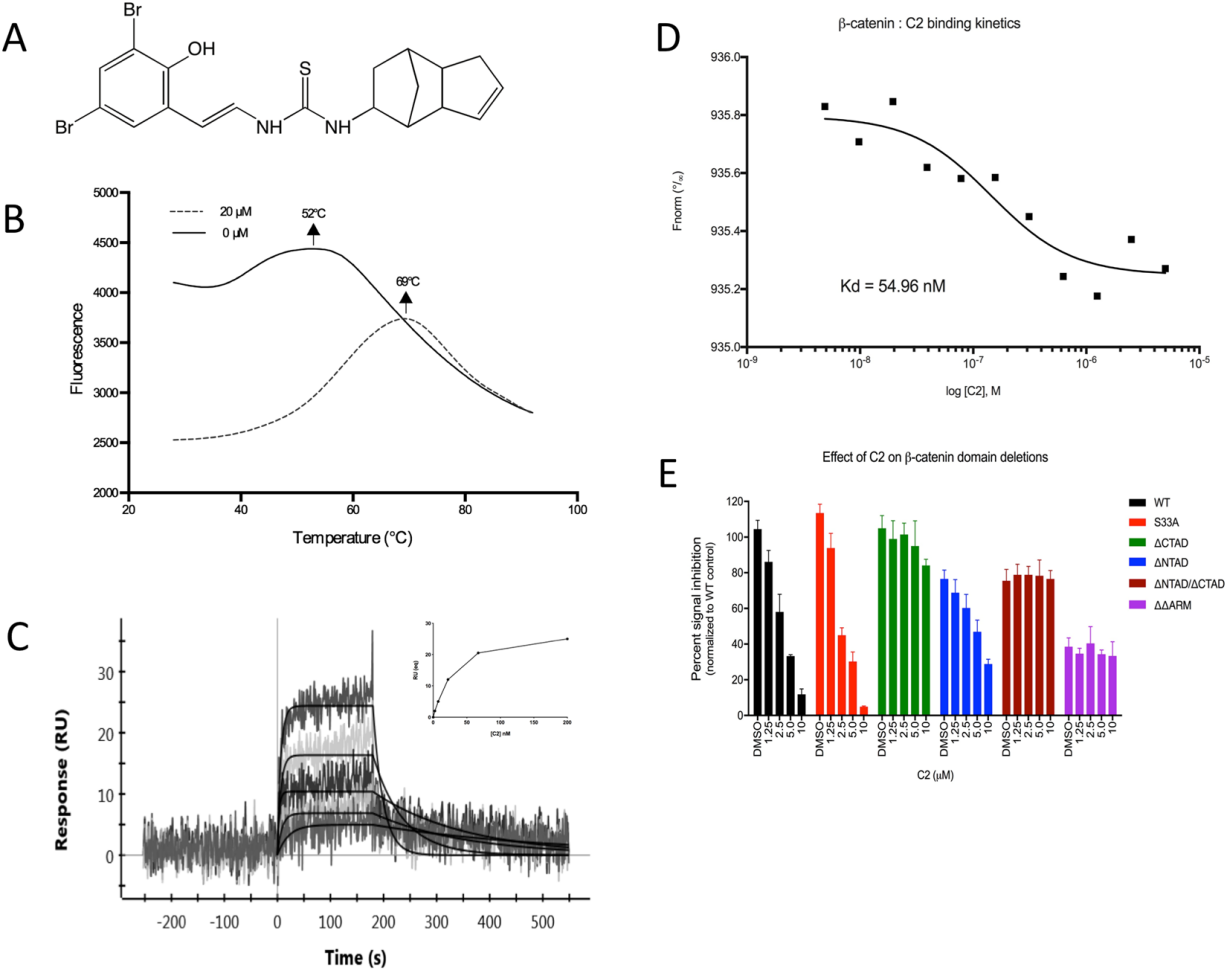
Biophysical characterization of C2. (**A**) Molecular structure of C2. Molecular weight: 484 [g/mol]; molecular formula: C19H20Br2N2OS; SMILES: N(C(=S)NC=Cc1cc(Br)cc(Br)c1O)C2CC3CC2C4C=CCC34. (**B**) Thermal melting profile of 2.5 μM purified wild-type human β-catenin protein in complex with 20 μM C2. (**C**) Binding sensorgram of C2 to β-catenin as measured with surface plasmon resonance (SPR). Five doses of C2 (12, 37, 110, 330 and 1000 nM) were injected over immobilized β-catenin. Association and dissociation rate are indicated. Jagged lines represent sensorgram data for injections of C2. Smooth lines represent fitting to Langmuir kinetic model. The estimated kinetics constants: K_on_ = 42.37 × 10^5^ M/s; K_off_ = 6.84 × 10^−3^ 1/s; K_d_ = 2.89 × 10^−8^ M. (**D**) Binding kinetics data of β-catenin with C2 measured with Micro-scale thermophoresis (MST). Various concentrations of C2 ranging from 0 to 20 µM with 2-fold dilution series were tested in duplicate. The binding signal from a reference flow capillary containing no protein was subtracted to account for detection of specific interaction between the compound and β-catenin. (**E**) Domain deletion reporter for β-catenin. 10 signaling pathways (WNT, Notch, p53/DNA damage, TGFβ, cell cycle, NFκB, Myc/Max, HIF-1, MAPK/ERK and MAPK/JNK) along with positive and negative controls were tested. All measurements were done in triplicates (n = 3).

Screening of NCI DTP library of 175,000 compounds against site C yielded a refined list, of which a list of 16 compounds was tested in the TopFlash luciferase reporter assay (Supplementary Fig. 2, Supplementary Table 1). Based on reporter ranking, the compounds were classified into two categories as inhibitors (C2, C11 and C16) and activators (C1, C3, C4, C5, C6, C7, C8, C9, C10, C12, C13, C14, C15). Intriguingly, under the given assay conditions these inhibitors had a stronger inhibitory effect than iCRT3 and StAx35. In a further TopFlash/FopFlash reporter assay, we tested dose range of C2, C11 and C16, in order to directly compare the inhibitory effect on both Top and Fop components (Supplementary Fig. 3). While all three compounds had comparable inhibitory effect on the TopFlash reporter above 2.5 µM, compounds C11 and C16 also negatively affected the FopFlash reporter, thus indicating potential cell toxicity. Therefore, we selected compounds C2 (NSC 211416) (Fig. 1A) as the strongest and less toxic candidate. In a further protein thermal shift assay, we confirmed target engagement of (Fig. 1B). In its native form, thermal melting of β-catenin revealed a highly flexible state represented by a broad peak at 52 °C, which is consistent with intrinsic disorder of full-length β-catenin protein. Addition of 20 µM C2 triggered efficient conversion of native β-catenin protein conformers into significantly more stable and more folded conformer in complex with C2, which melted at 69 °C. We subsequently measured binding affinity of C2 to β-catenin using surface plasmon resonance (SPR). We were able to register dose-dependent response and a binding affinity of 29 nM where the association and dissociation rates were typical of a small molecule lead candidate (Fig. 1C). We hence decided to focus our further efforts on candidate C2, which interacted with β-catenin with a nanomolar binding affinity.

In order to confirm binding kinetics between β-catenin and C2, we used an independent method, Micro-scale thermophoresis (MST). This method relies on the hydration shell, charge or size of molecules by measuring changes of the mobility of molecules in microscopic temperature gradients^24^. Purified HIS-tagged β-catenin protein was used, and eight concentrations of C2 ranging from 0.0012 to 20 µM with 2-fold dilution series were tested in triplicate. We recorded a dose-dependent response of C2 with β-catenin, with K_D_ calculated at 54.96 nM (Fig. 1D and Supplementary Fig. 4). This closely matches with the binding affinity data from SPR with only 2-fold difference.

Based on *in silico* approximations, we estimated the binding site of C2 to be in the junction of Armadillo domain and C-terminal Tans-Activation Domain (CTAD), spanning between residues 531–722. Therefore we questioned domain-specific interaction of C2 with β-catenin, in order to gain deeper insight into its molecular mechanism. We designed several TopFlash constructs carrying various domains of β-catenin, particularly Wild-type (WT), S33A, ΔNTAD, ΔCTAD, ΔNTAD/ΔCTAD and ΔARM (Supplementary Fig. 5). The latter three constructs are especially designed to dissect Site C, thus rendering interaction with C2 impossible. In order to make direct comparison between the different constructs, we ensured that transfection efficiency and starting cell viability was comparable between the constructs (Supplementary Fig. 6). Subsequently, Hek293 cells transfected with these reporters were treated with a dose range (10 µM–1.25 µM) of C2 for 24 hours and corresponding luciferase signal was quantified (Fig. 1E). We found that reporters WT, S33A and ΔCTAD had the highest level of starting signal. Only reporters WT and S33A responded to C2 in dose dependent manner, with EC50 around 2.5 µM, whereas reporter ΔCTAD largely lacked response. Even though reporter ΔNTAD started with 20% lower signal intensity, it was responsive to C2 in dose-dependent manner, with EC50 between 2.5–5.0 µM. Reporter ΔNTAD/ΔCTAD also started with 20% lower signal and was not responsive to C2. Finally, reporter ΔARM had the least starting signal intensity, approx. 60% less than WT control, and was similarly not responsive to C2 treatment. Such differential effect demonstrates that CTAD region is required for the dose-dependent effect of C2, suggesting that C2 binds to the CTAD domain, most likely at the proximity with ARM domain of β-catenin. The various levels of response obtained from these constructs is reflective of their corresponding contribution to β-catenin oncogenicity, and is also in line with previous reports^25^.

We subsequently measured binding affinity of C2 to β-catenin domains (ΔNTAD, ΔCTAD, ΔNTAD/ΔNTAD and ΔARM) using SPR. We were able to measure binding affinity for each domain: 11 nM, 12 nM, 14 nM and 108 µM for domains ΔNTAD, ΔCTAD, ΔNTAD/ΔNTAD and ΔARM, respectively (Supplementary Fig. 7). As expected, domain ΔARM had 10.000-fold lower affinity than the other domains. This data ultimately proved that C2 bound directly to ARM domain of β-catenin, and required this domain for high-affinity interaction.

### Selectivity for β-catenin

**Figure 2:**
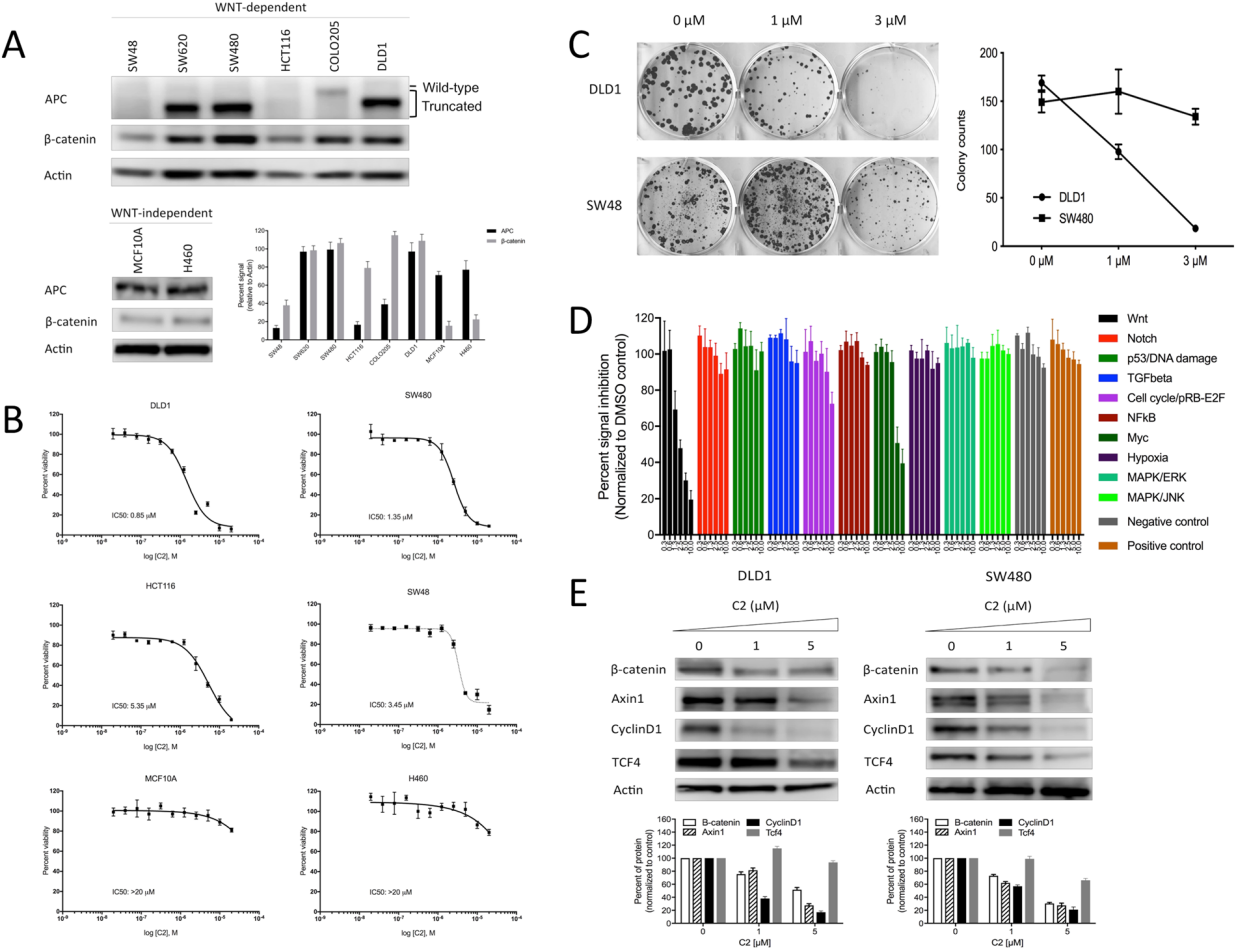
Selectivity of C2 for Wnt pathway. (**A**) Screening of β-catenin-dependent cell lines. (**B**) Effect of C2 on viability of colon cancer cell lines (24 hour treatment). IC50 values are shown for respective cell lines. (**C**) Colony assay for WNT-dependent vs independent cells (7 day treatment). The number of colonies in each well was counted after 7 days of incubation. (**D**) Cancer 10-pathway selectivity assay (24 hour treatment). (**E**) Western blot analysis of Wnt activity in DLD1 and SW480 cells (24 hour treatment). All measurements were done in triplicates (n = 3).

We focused on designing cellular assays that would selectively target β-catenin over-expression. We initially screened a panel of six WNT-dependent colon cancer cell lines together with two WNT-independent cell lines, in order to rationalize our selection of model for further studies (Fig. 2A, and Supplementary Fig. 8). As expected, we found that truncated APC in DLD1, SW480 and SW620 cells correlated with high β-catenin expression. On the other hand, wild-type APC levels in HCT116, SW48 and COLO405 cells correlated with lower β-catenin expression. Therefore, we chose to use DLD1 and SW480 cells to represent high β-catenin expression, and HCT116 and SW48 cells to represent low β-catenin expression. A total of six cell lines were then subjected to cell viability test, where an escalating dose range of C2 was applied (Fig. 2B). C2 reduced viability of DLD1 and SW480 cells in dose-dependent manner with IC50 ranging between 0.8–1.3 µM. Interestingly, viability of HCT116 and SW48 cells was impacted at higher C2 concentrations, with IC50 3.45–5.35 µM. Hence we observed 3-to-5-fold difference in viability between high and low β-catenin expressing cells. The two WNT-independent cell lines, MCF10A and H460, were only responsive above 10 µM. To further confirm this selectivity, we performed colony assay using DLD1 and SW48 cells (Fig. 2C). Expectedly, C2 inhibited the colony forming ability of DLD1 by 2-fold at 1 µM and almost completely eliminated it at 3 µM, whereas SW48 was only partially affected at 1 µM. This data was sufficient to demonstrate selectivity of C2 on β-catenin-overexpressing cells.

We further questioned selectivity of C2 to WNT pathway and other signaling pathways. We used Cignal Finder Cancer 10-Pathway Reporter Array to interrogate effect of C2 on various cancer signaling pathways. Hek293 cells transfected with the reporters were treated with a dose range (10 µM–0.3 µM) of C2 for 24 hours. Efficiency of transformation and cell viability was checked across all reporters and was found to be consistent (Supplementary Fig. 9). Candidate C2 inhibited signal of WNT pathway, while it mostly discriminated against the other reporters (Fig. 2D). We found that WNT pathway was the most responsive to C2 treatment, with EC50 around 2.5 µM. Considering that this is a non-physiological reporter system, we found this response concomitant with the subsequent viability assays. Transformation of cMyc and cell cycle pRB-E2F reporter was reduced to a minor extent, an observation that can be explained with their direct interaction with β-catenin and their role in cell cycle. We were convinced that the inhibitory effect of C2 was selective to WNT pathway via direct interaction with β-catenin.

To confirm the effect of C2 on the protein level, we tested for several markers of Wnt pathway in DLD1 and SW480 cells (Fig. 2E, also Supplementary Fig. 10) after 24 hours of treatment with C2. We observed similar patterns in both cell lines. Levels of β-catenin, Axin1, CyclinD1 and TCF4 were reduced in dose-dependent manner, indicating the effect on β-catenin nuclear complex and cell cycle, and in line with previous reports^20,26–28^. We thus concluded that C2 selectively inhibited β-catenin in Wnt signaling.

### Degradation of β-catenin

**Figure 3:**
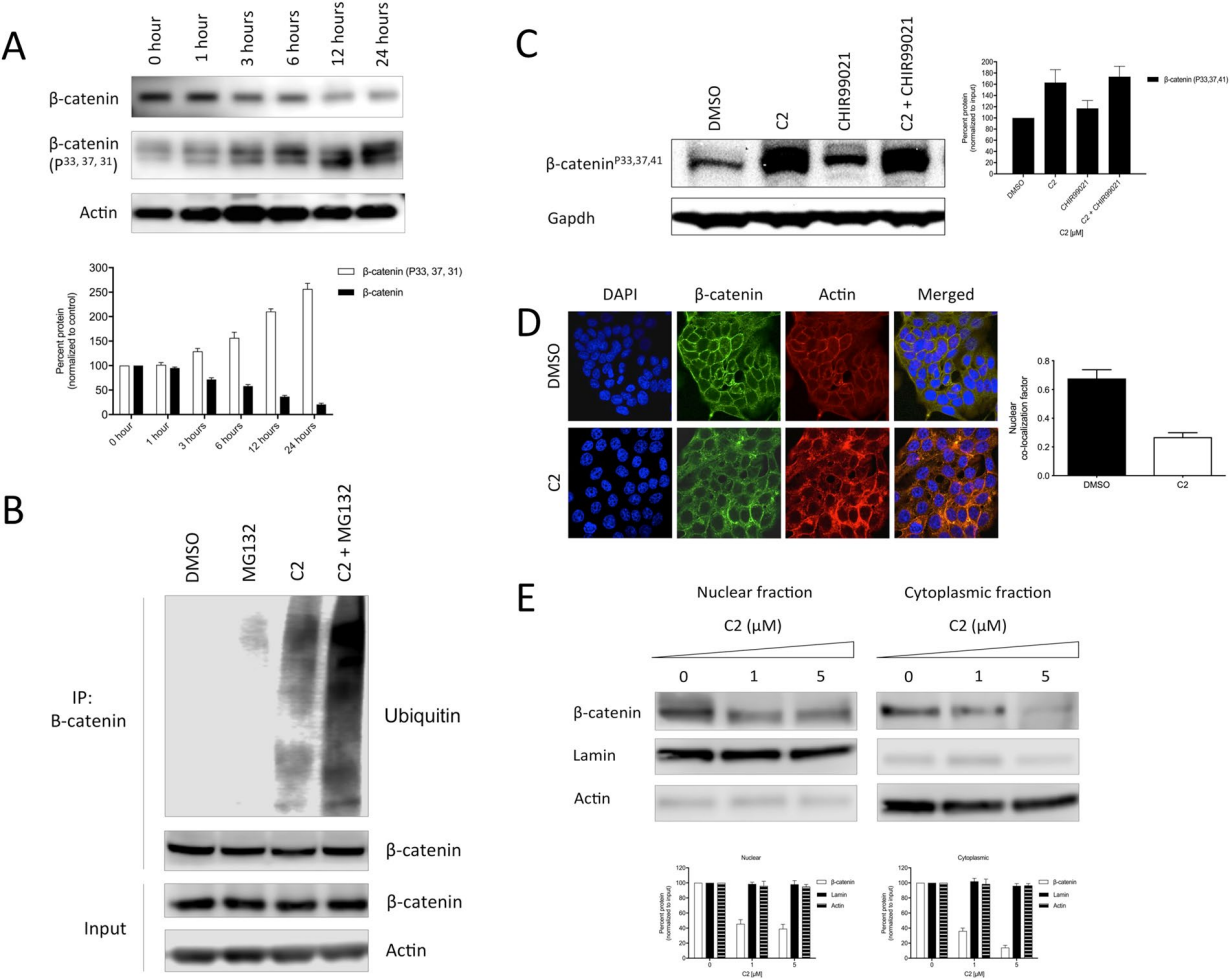
Mechanism of action of C2. (**A**) Time-course analysis of phosphorylation of β-catenin (1 µM C2). (**B**) Effect of C2 on ubiquitination of β-catenin (3 hour treatment). MG132 was used as proteasome inhibitor. (**C**) Combination of C2 (1 µM) with GSK3β inhibitor CHIR99021 (1 µM) (3 hour treatment). (**D**) Cellular localization of β-catenin as measured by confocal microscopy (24 hour treatment). Co-localization factor represents the level of β-catenin overlapping with nuclear DNA. Co-localization factor was calculated as 0.62 ± 0.03 and 0.25 ± 0.15 for DMSO and C2 treatment, respectively. (**E**) Nuclear-cytoplasmic fractionation of cells treated with C2. All measurements were done in triplicates (n = 3).

Understanding the mechanism of action of C2 is the first step towards improving its pharmacology. We initially set up a time-course analysis to capture the phosphorylation and degradation events of β-catenin. DLD1 cells treated with 1 µM of C2 were immuno-blotted at 1, 3, 6, 12 and 24 hour time points (Fig. 3A, also Supplementary Fig. 11). We were able to observe early sings of phosphorylated (at residues 33, 37, 31) β-catenin starting at 1 hour point, which was further amplified to 2–2.5-fold at 12 and 24 hour points. In line with this, levels of active β-catenin markedly reduced at these time points and recorded the lowest level at 24 hour point. Such clear phosphorylation event marked the essential component of the cascade for degradation of β-catenin.

We further set to test the effect of C2 on degradation of β-catenin through canonical ubiquitin-proteasome pathway, an established recycling mechanism for β-catenin^8^. We used proteasome inhibitor MG132 to block proteasomal processing and accumulate ubiquitinated β-catenin for immuno-precipitation (Fig. 3B, also Supplementary Fig. 12). At 3 hour time point, treatment with C2 showed increase in molecular weight of immune-precipitated β-catenin, reflecting the phosphorylation events observed earlier. Combination of C2 with MG132 arrested ubiquitination of β-catenin, suggesting additive proteosomal effect. Altogether, our data demonstrated that treatment by C2 triggered degradation of β-catenin through ubiquitin-proteasome machinery, presumably via recruitment of the APC-mediated destruction complex.

To further decipher the mechanism of action of C2, we questioned the role of phosphorylation events that are known to regulate the function of β-catenin^2,6,7^. We combined C2 with CHIR99021, a known inhibitor of GSK3β, to modulate the phosphorylation patterns of β-catenin (Fig. 3C, also Supplementary Fig. 13). Treatment with CHIR99021 alone inhibited GSK3β, which in turn blocked phosphorylation at N-terminus of β-catenin. Treatment with C2 alone, on the other hand, had an expected stimulating effect on its phosphorylation. Interestingly, when combined, C2 negated the effect of CHIR99021 and eventually increased phosphorylation of β-catenin. These results demonstrate that the mechanism of action of C2 involves stimulating the various phosphorylation events that drive turnover of β-catenin.

Next, using confocal microscopy, we visualized the distribution pattern of β-catenin in DLD1 cells (Fig. 3D and Supplementary Fig. 14) in response to 24-hour treatment with C2. We observed high abundance of β-catenin in the cell membrane as well as internal compartments. Using differential staining, we able to measure that level of β-catenin that co-localized with nucleic acids inside the nucleus. Upon treatment with C2, the overall β-catenin staining was reduced by 55-60% (co-localization dropped from 0.62 to 0.25), while its abundance was limited to the cell the cell surface. This data suggested that C2 reduced overall cellular concentration of β-catenin, except in the cell junctions.

Depletion of β-catenin in the cytoplasm and nucleus was confirmed via nuclear-cytoplasmic fractionation of DLD1 cells. Cells treated with C2 at concentrations 1 µM and 5 µM for 24 hours showed dose-dependent reduction of β-catenin over the course of 12 hours (Fig. 3E and Supplementary Fig. 15). At 1 µM, levels of β-catenin were reduced to 45% and 40% in the nuclear and cytoplasmic fractions, respectively. At 5 µM, the levels further reduced to 40% and 15%, respectively. C2 clearly impacted cellular load of β-catenin and thus encouraged its further *in vivo* study.

### Effect on organoid clonogenicity

As already acknowledged, truncated or otherwise mutated *APC* will lead to high β-catenin expression and thus drive proliferation of colon cancers^29,30^. We used *LGR5*^+^ *GFP*^hi^ intestinal organoid cultures derived from *APC*^fl/fl^ *LGR*5-EGFP-cre-ERT2 mice, as previously described^31^. We tested the potency of C2 on crypt stem cells in organoid culture, a novel surrogate method to study tumorigenesis of the colon (Supplementary Fig. 16). In comparison to wild-type, *APC*-deleted crypt appeared significantly larger and displayed aggressive growth. We found that C2 inhibited APC-deficient organoid clonogenicity in a dose-dependent manner. At 3 µM and 10 µM, viability of *APC*-deleted averaged around 70% and 55%, respectively. On the contrary, viability of wild-type organoids was 90% at the highest dose of C2. Thus we were able to demonstrate that *APC*-deleted colon organoids were notably more sensitive to C2 than their wild-type counterparts.

### *In vivo* mouse model

**Figure 4:**
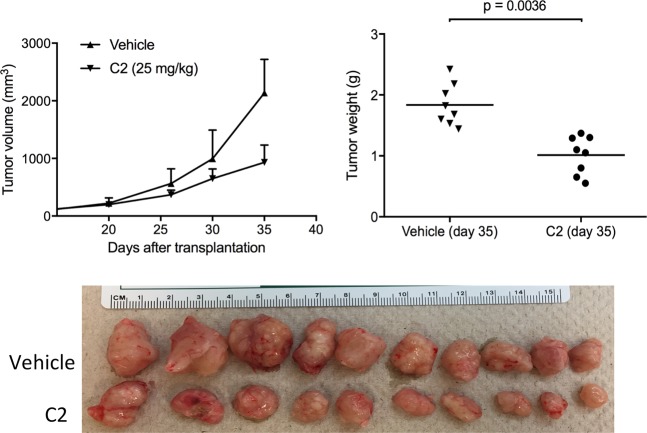
Effect of C2 on tumor growth. (**A**) Mouse xenograft test. Tumor volume (P = 0.00036), tumor growth (P = 0.0017) and actual images of tumors are shown. Error bars are in SEM.

Thus we were able to demonstrate that C2 had inhibitory effect on the growth of cancer-initiating cells driven by APC mutation. We next pursued mouse xenograft experiments using DLD1 cells (Fig. 4A). Mice treated with 25 mg/kg of C2 did not display any signs of acute toxicity and body weight loss. Inhibition of tumor growth by C2 was noted after 20 days of treatment, after which the tumor volume of C2-treated mice did not increase exponentially. Upon surgery on day 35, the weight of the tumors corresponded to their respective volumes. At day 35, the calculated tumor growth inhibition by C2 was 50% as compared to the vehicle. These observations suggest that C2 notably reduced β-catenin-dependent tumor growth represented in DLD1 xenograft model. Our data suggest sufficient bioavailability of C2 *in vivo* with promising therapeutic properties.

## Discussion

Uncontrolled expression of β-catenin is known to drive multiple types of cancers, including colon cancer, hepatocellular carcinoma and melanoma^10–14^. There is an increasing body of evidence suggesting that inhibition of β-catenin can suppress tumor progression as well as recurrence^17^. Involvement of β-catenin in healthy tissue homeostasis and activating mutations that render β-catenin refractory to proteosomal degradation are just two factors that make pharmacological targeting of β-catenin challenging^1–3^. Therefore, much recent effort has been spent of direct targeting of β-catenin, which is acknowledged to have the most therapeutic relevance. However, there is still a need for novel mechanism-based inhibitors that directly target active β-catenin in cells that are driven by its over-expression. This is a challenging task, since the roles of β-catenin in health and disease are tightly interwoven, and sensitive screening and assay methods are required for identification of selective inhibitors^21^.

In the current study, we report discovery of C2, an experimental small-molecule inhibitor of β-catenin. C2 targets an allosteric site on the surface of β-catenin, presumably alters its conformational state and thereby triggers its degradation via proteasome system. In its initial chemically un-optimized form, C2 selectively reduced viability of β-catenin-driven cancer cells, which harbor *APC* mutations. It is particularly remarkable that C2 can engage its target in the cells and thereby trigger its degradation. Over-expression of β-catenin is typical for many colon cancers, in which β-catenin accumulates on oncogenic levels by becoming refractive to degradation. The ability to deplete excessive β-catenin to degradation makes C2 a promising inhibitor, which has a clear advantage over less selective inhibitors that target the upstream partners of Wnt pathway.

Some of the previously reported β-catenin inhibitors are believed to target its nuclear fraction and thereby stop its oncogenic activity^18,20^. C2, on the other hand, targets the general cellular pool of β-catenin, which reflects onto both cytoplasmic and cellular compartments, as demonstrated by our fluorescent microscopy data. We believe that C2 targets free β-catenin inside the cell, removes it from circulation in the cell, and thereby reduces its overall active concentration – a process that in turn halts its oncogenic role. We have shown that treatment with C2 stimulates its phosphorylation and primes it for ubiquitination for its ultimate degradation. Even though the exact mechanism of action of C2 is yet to be established, our study demonstrates that C2 has the potential to evolve into a mechanism-driven therapeutic inhibitor of β-catenin. Our data cannot exclude the possibility that high doses of C2 might disrupt β-catenin in cell-cell junctions and lead to potential cell toxicity. Therefore, additional studies should address the potential toxicity of this experimental drug before it could be considered for potential clinical development.

β-catenin is known to interact with a large pool of binding partners from various functional families^5,32,33^. According to our computations, C2 targets the allosteric site C, which resides in armadillo domain 8–10 spanning between residues 531–722. This domain serves as an interaction platform for non-canonical binding partners of β-catenin, such as Hif1α, which is involved in adapting cancer cells to sugar metabolism^34,35^. One can postulate that targeted disruption of this relatively mutation-free interface would disrupt interaction between β-catenin and its oncogenic partners, and would thus provide a novel pharmacological approach for selective elimination of β-catenin-driven oncogenicity. This would require an inhibitor that can overcome binding affinity barriers in upwards of 30 nM in order to specifically disrupt protein-protein interactions in this domain^22,23^. Indeed, our SPR data demonstrate that C2 binds to β-catenin of 29 nM, with association and dissociation rate of a promising lead inhibitor that can overcome the energetic barriers inherent to β-catenin. Our mechanism-of-action data demonstrated that C2 was able to reduce oncogenic load of β-catenin in the cell and selectively inhibit cancer cell growth. These data served as a gateway to subsequent *in vivo* studies, which confirmed anti-tumor activity as well as sufficient bioavailability of C2. Even though we demonstrate that C2 can selectively target APC-driven colon cancer growth, subsequent more comprehensive studies will provide a detailed mechanistic rationale for its further optimization.

Future mechanistic studies and structure-activity optimization will need to address suitability of C2 as a clinical drug candidate. Here we report a novel pharmacologic approach for selective inhibition of β-catenin via a cryptic allosteric site. Further mutagenesis and crystallography studies of β-catenin – C2 complex need to elucidate the coordinates of Site C together with the precise binding interactions of C2. Mechanistic studies also need to address the effect of C2 on the thermodynamic phase transition of β-catenin, as a crucial step that leads to disruption of interaction with partner proteins such as Axin1/2. In this report, we demonstrate that it is feasible to identify novel mechanism-driven small-molecule inhibitors to β-catenin if selective screening and assay methods are assembled. Altogether, our findings provide a new perspective for therapeutic targeting of β-catenin.

## Methods

### Molecular modeling

We used Q-MOL molecular modeling software from Q-MOL LLC (San Diego, CA, USA; www.q-mol.com) for all molecular modeling, virtual ligand screening, cheminformatics and other computational biology tasks. All modeling and simulations were performed on a single desktop machine running Debian Squeeze Linux system (AMD FX-6100 six cores, 16 GB RAM), and in Q-MOL cloud services.

### Prediction of allosteric sites

Q-MOL molecular surface scanning methodology was used to identify putative allosteric hotspots. Briefly, minimized structures of characterized β-catenin inhibitors iCRTs^18^, and structures of 20 natural individual amino acids (treated as small molecules), were used as molecular probes to systematically scan the molecular surface of β-catenin by applying protein-ligand docking methodology as implemented in the Q-MOL molecular modeling platform (Supplementary Fig. 1). The surface scanning by a molecular probe allows determining excess of energy on the surface of a protein, stored in the form of unrealized or inefficient interactions among surface amino acid residues by probing interactions with these amino acid residues. In the case when free amino acid structures were used as probes, each of the 20 amino acids was individually docked across molecular surface of the target protein. Then amino acids were ranked by their specificity, and scan results were grouped for top 5 amino acids probes. This technology allows detecting and visualizing of allosteric and cryptic binding sites, binding sites of known hits, and protein-protein interaction interfaces. Upon completion of the surface scan, energy values were normalized and converted into probabilities of probe binding to a particular surface spot. For the purpose of the data analysis, these binding probabilities were visualized as 3D objects across the molecular surface of the β-catenin (Supplementary Fig. 1).

### Virtual ligand screening

Virtual ligand screening (VLS) was performed as previously described^36–38^. The ligand docking simulations were performed using the β-catenin crystal structure coordinates from PDB 2GL7 (chain A). BCL9 and TCF4 peptides were removed from the complex before docking simulations. The protein molecule preparation included adding of hydrogen atoms and the assignment of the OPLS atom types. The ligand-binding center for site C (Supplementary Fig. 1) was defined within the radius of 10 Å of residues P520, R527 and D582. Upon completion of VLS, the initial hits were visually inspected, and available compounds were ordered from the NCI Developmental Therapeutics Program (NCI DTP) collection.

### Modeling of protein-ligand complexes

The predicted binding modes of the *in vitro* validated ligands were built using full-atom flexible protein-ligand docking in the internal coordinates as implemented by the Q-MOL program. Briefly, the initial ligand conformations were taken from the VLS experiments. The protein-ligand complex was then globally optimized in the OPLS^39^ force field using the Monte Carlo simulation in internal coordinate space. The protein *Phi*, *Psi* and *Xi* angles were allowed to change. In the case of a ligand molecule, its positional and rotable torsion variables were unfixed.

### TopFlash/FopFlash luciferase reporter

Selected compound candidates were obtained from the NCI DTP; 20 mM stocks were prepared in DMSO. HEK293 cells were seeded in 6-well plates and transfected with a cocktail of the following plasmids: Cells were cultured in DMEM and 2% FBS at 37 °C for 1 day, then transferred into 96-well plate format (10.000 cells per well). After 12 hours, DMEM medium with diluted compounds was added to the cells. Initially, single dose (3 µM) per compound. Similarly, for TopFlash/FopFlash assay, HEK293 cells were transfected with a cocktail of these plasmids: 1 µg FopFlash, 1 µg pcDNA3-β-catenin and 50 ng pRL-TK using Lipofectamine 2000 (Invitrogen). For this assay, a dose range between 20 µM and 0.3 µM was tested for each compound. Luciferase reporter activity was measured using the Dual-Glo system (Promega) after 24 hours of incubation. Relative luciferase activity is normalized to DMSO control. All measurements were done in triplicates (n = 3).

### Domain deletion assay for β-catenin

Domains of β-catenin were cloned into pcDNA3 plasmid using primers described before^25^, and the final constructs were confirmed with sequencing. HEK293 cells were cultured and transfected using Lipofectamine 2000 with a cocktail of these plasmids: 1 µg TopFlash, 1 µg of corresponding pcDNA3 construct and 50 ng pRL-TK. Cells were treated with a dose range (10–1.25 µM) of C2, incubated for 24 hours, then luciferase signal was measured. Signal was normalized to DMSO treatment of WT control. All measurements were done in triplicates.

### Protein expression and purification

BL21 cells harboring the pPET28a-TEV-full-length human β-catenin (Addgene) plasmid were cultured at 37 °C until OD600 = 0.8 and were then induced with 1 mM isopropyl-β-D-thiogalactopyranosid (IPTG). Cells were cultured for another 5 h at 20 °C. Cell pellets were collected and sonicated in buffer with 300 mM NaCl, 20 mM Tris (pH 8.8). Soluble His-tagged β-catenin was purified from cell lysate using a HiTrap column packed with Sepharose resin (GE Healthcare), with a gradient of 20–200 mM imidazole. Purified protein was dialyzed into the same buffer without imidazole. Domains of β-catenin (ΔNTAD, ΔCTAD, ΔNTAD/ΔNTAD and ΔARM) were cloned into the same plasmid. Expression and purification of these proteins were done using the same protocol.

### Thermal shift assay

Thermal shift assays (TSA) were conducted with the 96-well-based CFX-96 real-time fluorescence plate reader (BioRad, Hercules, CA). The fluorescent dye Sypro Orange (Sigma, St. Louis, MO) was used to monitor the protein folding-unfolding transition. Protein-ligand binding was gauged by shift in the unfolding transition temperature (ΔTm) acquired with protein alone or with protein in the presence of the inhibitor. 2.5 μM of purified wild-type human β-catenin protein was mixed with 20 μM of candidate compound in PBS buffer containing 2X SYPRO Orange dye. Negative control: protein plus corresponding amount of DMSO. Positive controls: StAx35R and iCRT3, 10 μM each. The sample plate was heated from 25 °C to 95 °C with a thermal ramping rate of 1 °C/min. The fluorescence signals were acquired with excitation and emission wavelengths centered at 490 and 560 nm, respectively. Differences in melting temperatures (ΔTm) were calculated via subtraction of Tm of the negative control.

### Surface plasmon resonance

Interaction of with C2 with His-tagged β-catenin and its domains was measured using surface plasmon resonance (SPR) on a ProteOn instrument (Bio-Rad). First, protein was captured on the activated surface of HTE chip. Binding experiments were run in PBS buffer supplemented with 0.01% P-20 and 1% DMSO. C2 was dissolved in the same buffer and passed over His–β-catenin functionalized surface as well as activated blank reference. Measurements were performed in triplicates with three-fold dilutions (0.2 µM to 0.0025 µM). Binding constants (K_on_ for association rate, K_off_ for dissociation rate) were calculated via fitting of data to 1:1 Langmuir model.

### Micro-scale thermophoresis

Binding Kinetics was measured for C2 and β-catenin using microscale thermophoresis. A range of eight concentration points of C2 (ranging from 0.0012 to 20 µM) was incubated with 50 nM purified β-catenin protein for 5 min in assay buffer (PBST with 2.5% DMSO). The samples were loaded into NanoTemper glass capillaries and micro-thermophoresis was carried out using 60% excitation power and 40% MST, 25 °C. K_D_ was calculated using the mass action equation via the NanoTemper software MO.Affinity Analysis v2.2.5 from duplicate reads of triplicate experiments. The instrument used was a NanoTemper Monolith NT.115.

### Cell viability assays

Fully tested and authenticated cancer cell lines (DLD1, SW480, SW48, SW620, HCT116, COLO205, MCF10A and H460) were purchased from ATCC (American Type Culture Collection). Cells were plated in 96-well plates with seeding density of 5000 cells per well in RPMI 1640 medium supplemented with 2% FBS. After 24 hours of incubation, a dose range of C2 under 20 µM was tested in triplicates. After 72 hours of incubator at 37 °C/and 5% CO_2_, cells were stained with Alamar blue and counted at wavelength of 560/590. Viability curves were plotted and IC50 values calculated using Prism8 GraphPad software.

### Colony assay for cancer cells

DLD1 and SW48 cells were plated in 6-well plates with seeding density of 400 cells per well in RPMI 1640 medium supplemented with 2% FBS. 24 hours later C2 (1 µM and 3 µM) or DMSO alone was added to the cells. Medium and treatment was renewed every other day. After 7 days of incubation, cells were stained with crystal violet and colonies were counted.

### Cancer 10-pathway reporter array

Signal Finder Cancer 10-Pathway Reporter Array included reporters for WNT (TCF/LEF), Notch (RBP-Jκ), p53/DNA damage, TGFβ (SMAD2/SMAD3/SMAD4), cell cycle (E3R/DP1), NFκB, Myc/Max, Hypoxia-inducible factor 1 (HIF-1), MAPK/ERK (Elk1/SRF), MAPK/JNK (AP1), together with negative control and positive control (Qiagen). All reporters carried luciferase construct with the corresponding transcription regulatory element together with constitutively expressing *Renilla* luciferase. Negative control carried a non-inducible firefly luciferase costruct and a constitutively expressing *Renilla* luciferase construct, while positive control carried a constitutively expressing luciferase construct and a constitutively expressing *Renilla* luciferase construct. HEK293 cells transfected with the reporters were treated with a dose range (10–0.3 µM) of C2 for 24 hours.

### Western analysis and immuno-precipitation

Cells were grown in RPMI 1640 medium supplemented with 2% FBS and 5 µM C2 or DMSO for 24 hours. For whole-cell western analysis, cell lysates were prepared in RIPA buffer (10 mM Tris-Cl (pH 8.0), 1 mM EDTA, 1% Triton X-100, 0.1% sodium deoxycholate, 0.1% SDS, 140 mM NaCl and 1 mM PMSF), and equal amount of cell lysate was loaded onto SDS-PAGE gel. Detection and quantitation of protein signal was done on LI-COR C-Digit Blot Scanner. For immuno-precipitation experiments, cells were treated with DMSO, C2 (5 µM), MG132 (10 µM) and combination of C2 (5 µM) and MG132 (10 µM) for 3 hours. Subsequently cells were lysed in TBS [150 mM NaCl and 20 mM Tris (pH 7.4)] with 1 mM PMSF and 1% Triton X-100 at 4 °C for 30 min, followed by removal of insoluble debris by centrifugation at 13,000 × g. Lysate with total protein amount of 500 µg was initially cleared with IgG, then incubated with 5 µg of anti-β-catenin antibody at 4 °C for 4 h. Then, protein A/G agarose beads (Santa Cruz Biotech) were added to the sample at 4 °C for 2 hours. Beads were washed 3× with TBS + 0.01% Tween 20, then boiled in SDS gel loading buffer at 95 °C for 5 min. Western blot analysis was performed as described above. 5% of total protein used for immune-precipitation was loaded as input. For time-course analysis, cells were treated with C2 at 1, 3, 6, 12 and 24 hour intervals, after which a standard western analysis protocol was applied. For nuclear-cytoplasmic fractionation, cultured DLD1 cells were resuspended in PBS buffer supplemented with 5% glycerol and protease inhibitors, then passed through 26 G needle multiple times on ice. Nuclear and cytoplasmic fractions were separated via centrifugation at 10.000 rpm. The fractions were then denatured in RIPA buffer and loaded onto SDS-PAGE gel for subsequent Western blot analysis. All primary and secondary antibodies were purchased from Cell Signaling Technologies.

### Confocal fluorescence microscopy

To investigate cellular distribution of β-catenin, HCT116 cells were seeded in chamber slides (LAB-TEK) and incubated in 0.5 ml RPMI 1640 medium supplemented with 2% FBS and 1 µM C2. After 12 hours of incubation at 37 °C, cells were washed with PBS and fixed with 4% paraformaldehyde in PBS. Cells were then probed with anti-β-catenin primary antibody (Cell Signaling), washed and then probed with Alexa Fluor-tagged anti-rabbit IgG secondary antibody (Life Technologies). Subsequently, cells were incubated with phalloidin rhodamine (Life Technologies). Finally, mounting media with DAPI was used to attach No 1.5 cover slides. Confocal fluorescence microscopy was performed with a Zeiss LSM 710 inverted confocal microscope [DAPI: λ(ex) = 420 nm, Alexa fluor: λ(ex) = 488 nm, phalloidin rhodamine: λ(ex) = 540]. The level of co-localization of β-catenin and DAPI was quantified using double-stained confocal sections (Alexa fluor/DAPI). Sections were processed using software packages ZEN lite and Fiji. To determine Pearson co-localization coefficient for twenty nuclei per sample, we used EzColocalization plugin for ImageJ image analysis software^40^. Co-localization of β-catenin and DAPI was compared for C2 and DMSO.

### Organoid clonogenicity assay

We derived LGR5^+^ GFP^hi^ intestinal organoid cultures from *APC*^fl/fl^*LGR*5-EGFP-cre-*ERT2* mice, as previously described^31^. To assess the ability of the inhibitor C2 to inhibit clonogenicity of *APC*-deficient intestinal organoids, we seeded 1000 live GFP^hi^ tumor stem cells into a three-dimensional collagen matrix (Matrigel), and then incubated with organoid growth media [ADMEM/F12, N2 (1:100), B27 (1:50)] containing C2 or DMSO. Four days later, clonogenicity was calculated as number of organoids per cell. Percentage viability of formed organoids was compared for C2 and DMSO.

### *In vivo* mouse model

6-week-old female NSG (NOD-SCID) mice were injected with 3 × 10^6^ DLD1 cells, resuspended in matrigel, into the left and right flanks. Xenografts were allowed to grow until the average tumor volume reached 100 mm^3^; then the mice were divided into two groups of six animals. One group received 25 mg/kg C2 and the other received vehicle (PEG400 + solutol + DMA) only. Doses were administered intra-peritoneally, and tumor volume and mouse weight were measured daily using external calipers. The mice were treated for a period of 35 days. At the end of the experiment (day 35), the mice were euthanized, tumors were excised, and tumors were weighed. All vertebrate experiments were conducted with the approval of Animal Care and Use Committee of University of California San Diego. Likewise, all vertebrate experiments were performed in compliance with guidelines set forth by the National Institute of Health and the Principles for the Use and Care of Vertebrate Animals.

## Supplementary information

Supplementary Materials.
