# Supplementary material for: Allosteric inhibitor of β-catenin selectively targets oncogenic Wnt signaling in colon cancer
Source: Sci Rep. 2020 May 15;10:8096. doi: 10.1038/s41598-020-60784-y (PMC7229215; doi:10.1038/s41598-020-60784-y)
Supplement: Supplementary file 1 — Supplementary Materials. [file 41598_2020_60784_MOESM1_ESM.pdf]

Allosteric inhibitor of β-catenin selectively targets oncogenic Wnt signaling in colon cancer

Anton Cheltsov [1], Natsuko Nomura [2], Venkata M Yenugonda [2], Jatin Roper [3], Rajesh Mukthavaram [4], Jiang Pengfei [4], Nam-Gu Her [5], Ivan Babic [2], Santosh Kesari [2], Elmar Nurmemmedov [2 *]

[1] Scripps Research Institute, Department of Molecular Medicine, La Jolla, CA, USA

[2] John Wayne Cancer Institute and Pacific Neuroscience Institute at Providence Saint John's Health Center, Santa Monica, CA

[3] The David H. Koch Institute for Integrative Cancer Research at MIT, Department of Biology, MIT, Cambridge, MA, USA

[4] Translational Neuro-Oncology Laboratories, Department of Neurosciences, University of California San Diego, La Jolla, CA, USA

[5] Korea Institute of Radiological and Medical Sciences, 75 Nowon-ro, Seoul 01812, Korea

[*] **Corresponding author**:

elmar.nurmammadov@providence.org

Running Title

Allosteric inhibitor of β-catenin

SUPPLEMENTARY FIRUGE LEGENDS

Supplementary Figure 1 - Molecular surface mapping of β-catenin**.** The molecular surface of β-catenin (PDB 2GL7) was mapped as described in Materials and Methods. The predicted allosteric hotspots are indicated as spheres. The size and color of spheres represent probability that predicted site is a ligand-binding site: red/big – high, blue/small – low. The locations of docking sites are indicated by circles and text labels. Blue helix – BCL9 peptide, yellow helix – LEF/TCF4 peptide.

Supplementary Figure 2 - TOPFLASH reporter ranking of hit compounds. Refined list of hit compounds targeting site C was subjected to TopFlash luciferase reporter assay as described in Materials and Methods. Compounds were ranked according to their percentage inhibition (10 µM) of luciferase signal, normalized to DMSO control. Negative ranking compounds are considered inhibitors, while positive ranking compounds are considered activators of β-catenin. iCRT3 and StAx35 are used as positive controls.

Supplementary Figure 3 – TOPFLASH and FOPFLASH luciferase reporter assays for inhibitors C2, C11 and C16. Compounds were tested for their ability to inhibit TOPFLASH reporter in dose-dependent manner without affecting FOPFLASH. C2 was thus selected as a selective inhibitor.

Supplementary Figure 4 – Quality control data for micro-scale thermophoresis (MST) measurements. Data shows capillary position, raw MST fluorescence and capillary shape at the time of measurements.

Supplementary Figure 5 – β-catenin domain deletion constructs prepared in pcDNA3 plasmid. These domains are Wild-type (WT), S33A, ΔNTAD, ΔCTAD, ΔNTAD/ΔCTAD and ΔARM. The constructs were used for domain selectivity reporter assay.

Supplementary Figure 6 – Transfection and viability control for the domain selectivity reporter assay. The data shows that transfection efficiency and cell viability is evenly distributed across the different constructs.

Supplementary Figure 7 – SPR data for C2 interaction with β-catenin domains ΔNTAD, ΔCTAD, ΔNTAD / ΔNTAD and ΔARM.

Supplementary Figure 8 – Un-cropped images of Figure 2A

Supplementary Figure 9 – Transfection and viability control for cancer 10-pathway reporter assay. The data shows that transfection efficiency and cell viability is evenly distributed across the different constructs.

Supplementary Figure 10 – Un-cropped images of Figure 2E

Supplementary Figure 11 – Un-cropped images of Figure 3A

Supplementary Figure 12 – Un-cropped images of Figure 3B

Supplementary Figure 13 – Un-cropped images of Figure 3C

Supplementary Figure 14 – Expanded confocal microscopy images from Figure 3D.

Supplementary Figure 15 – Un-cropped images of Figure 3E

Supplementary Figure 16 – Effect of C2 on viability of healthy and APC-/- organoids. The data shows that viability of APC-/- organoid is more notably affected than that of healthy organoids.

Supplementary Table 1 – List of 16 compounds selected for TopFlash reporter assay screening. Corresponding NSC numbers are shown.

Supplementary Figure 1

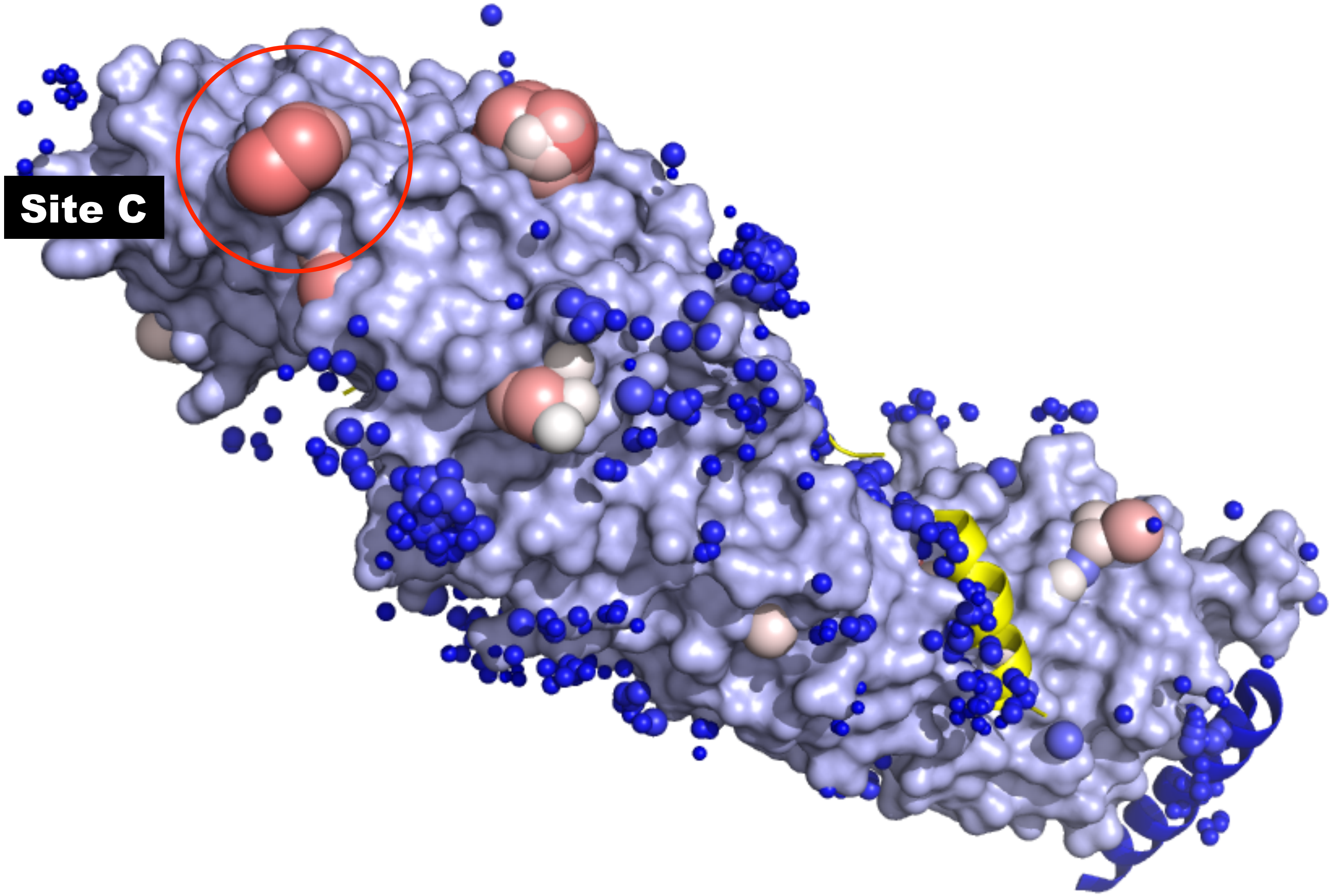

Supplementary Figure 2

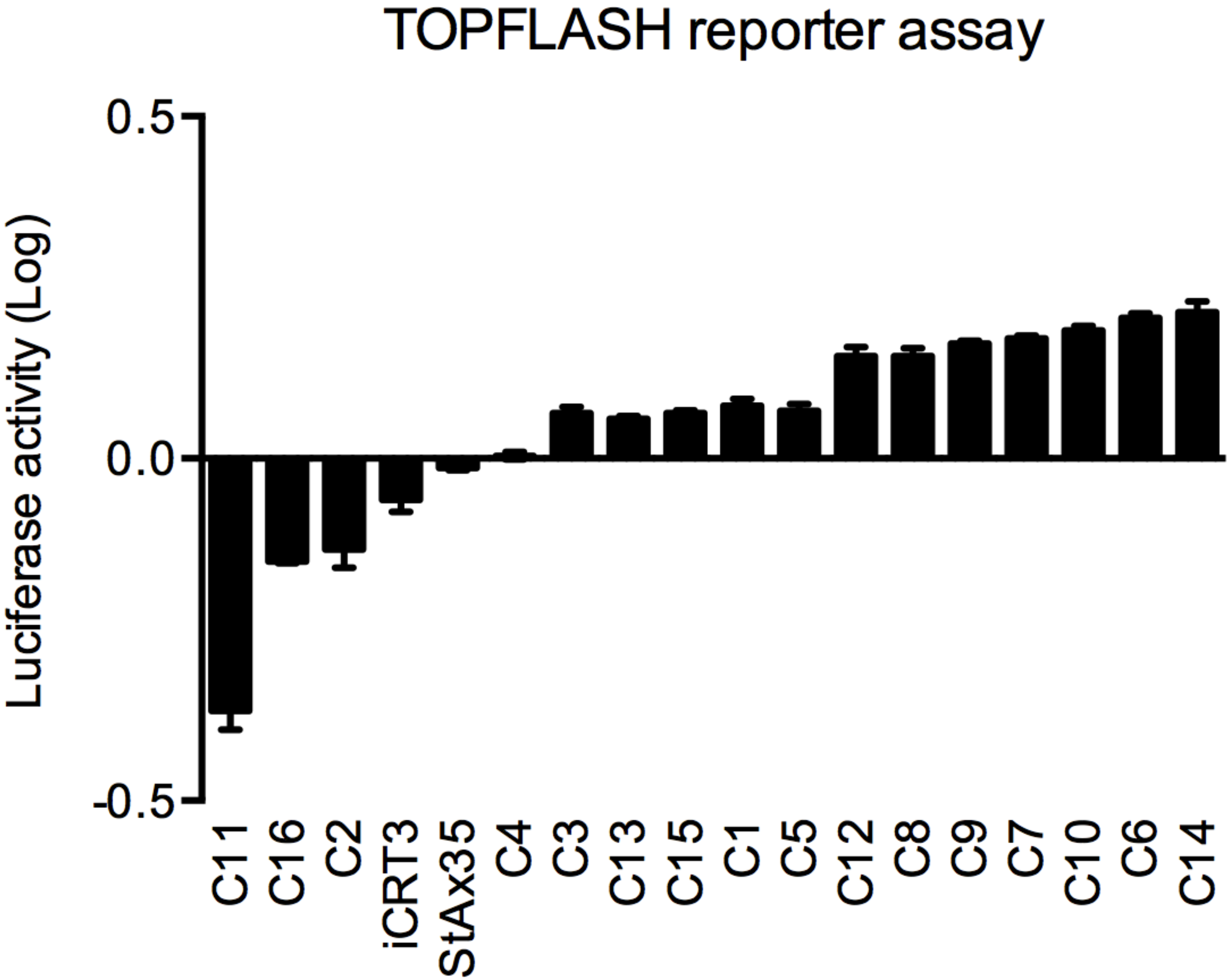

Supplementary Figure 3

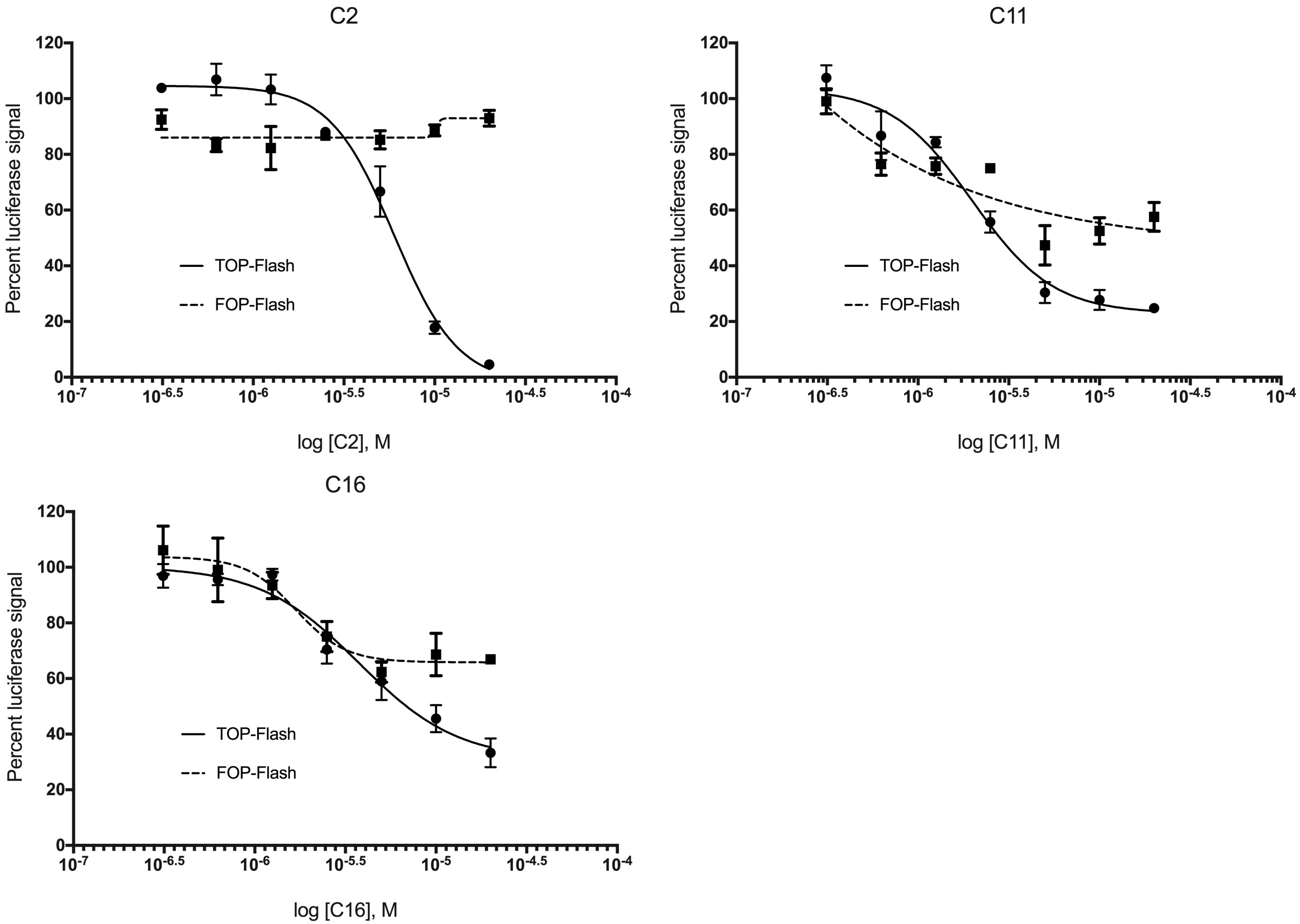

Supplementary Figure 4

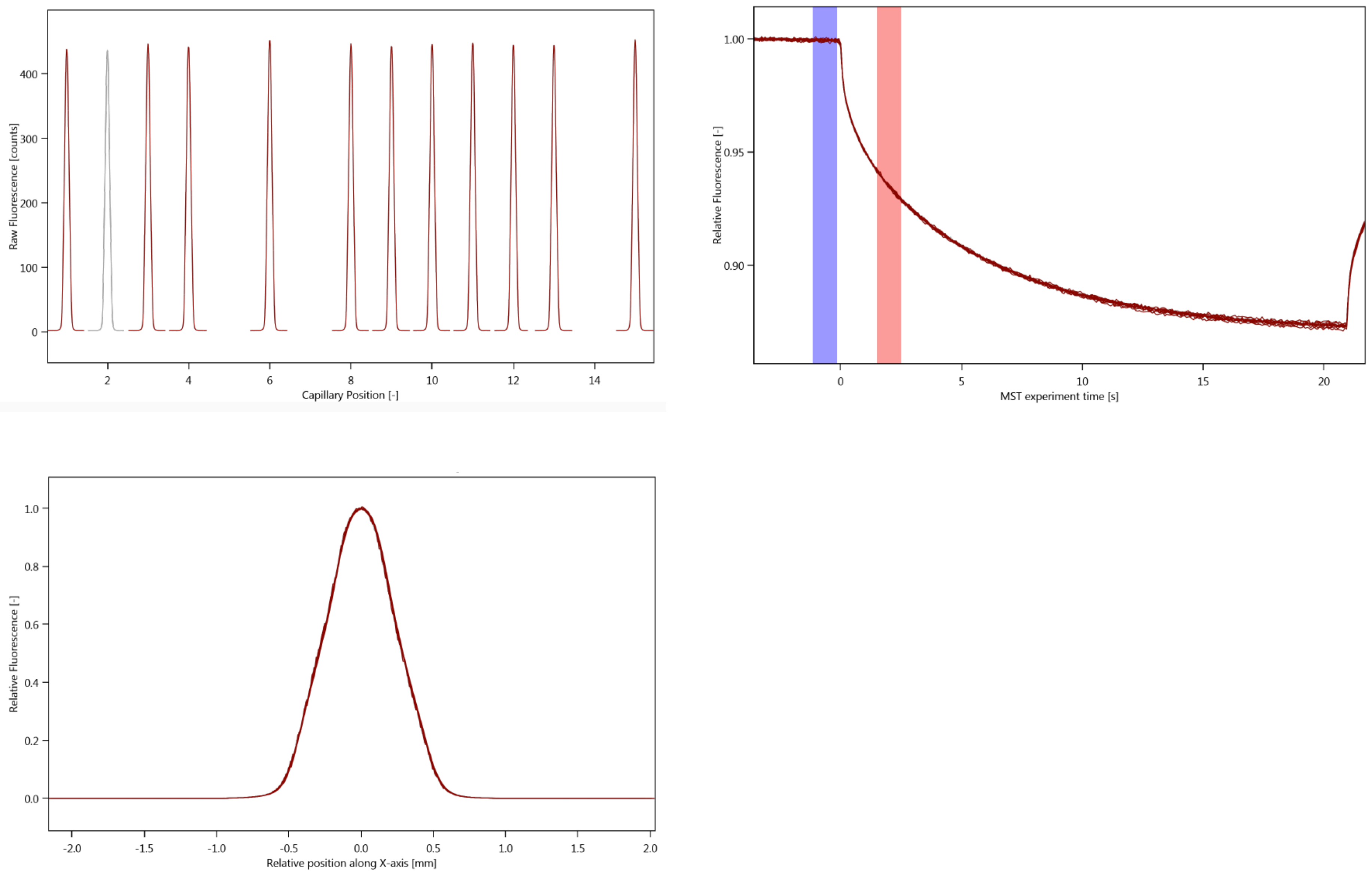

Supplementary Figure 5

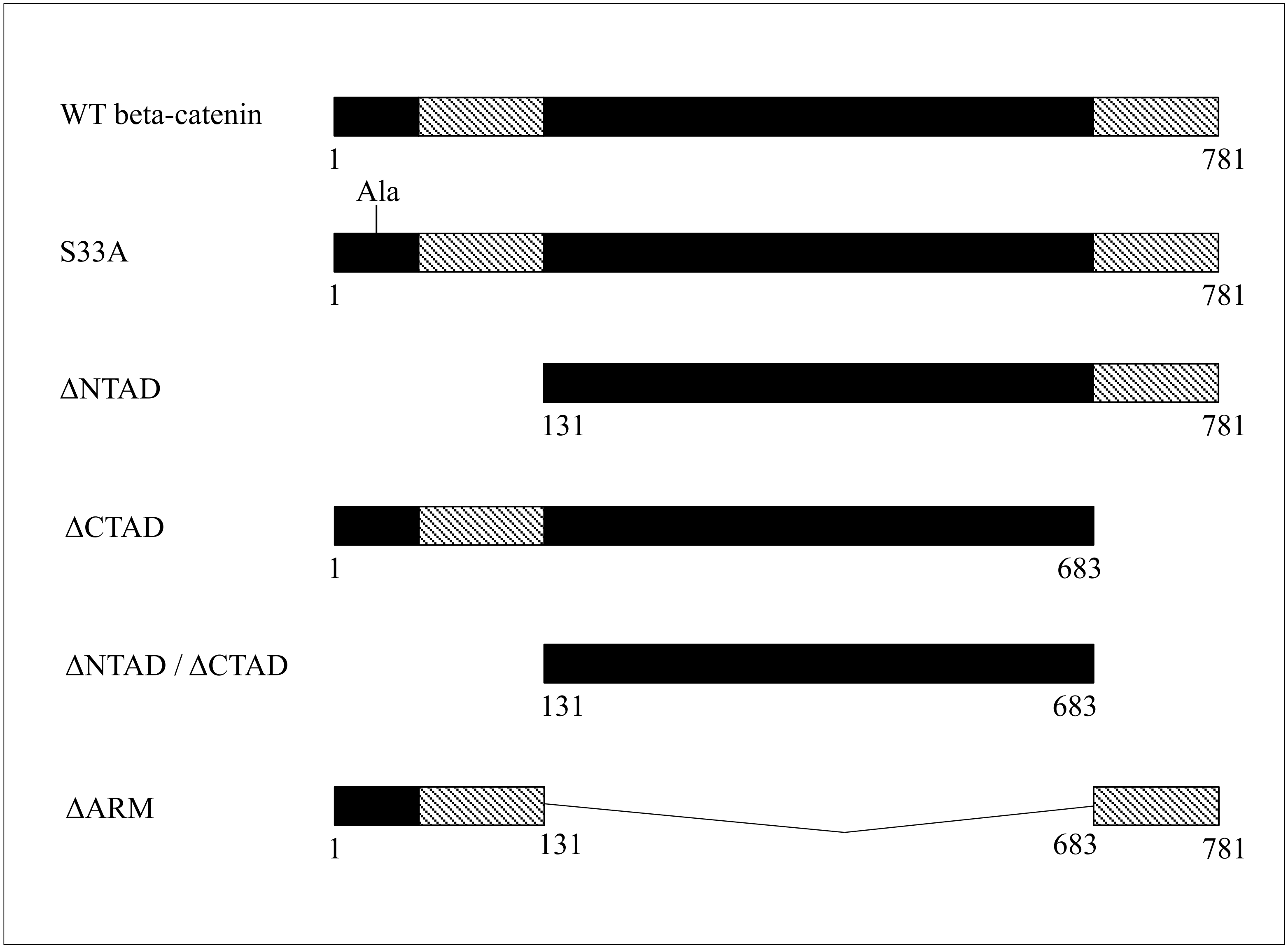

Supplementary Figure 6

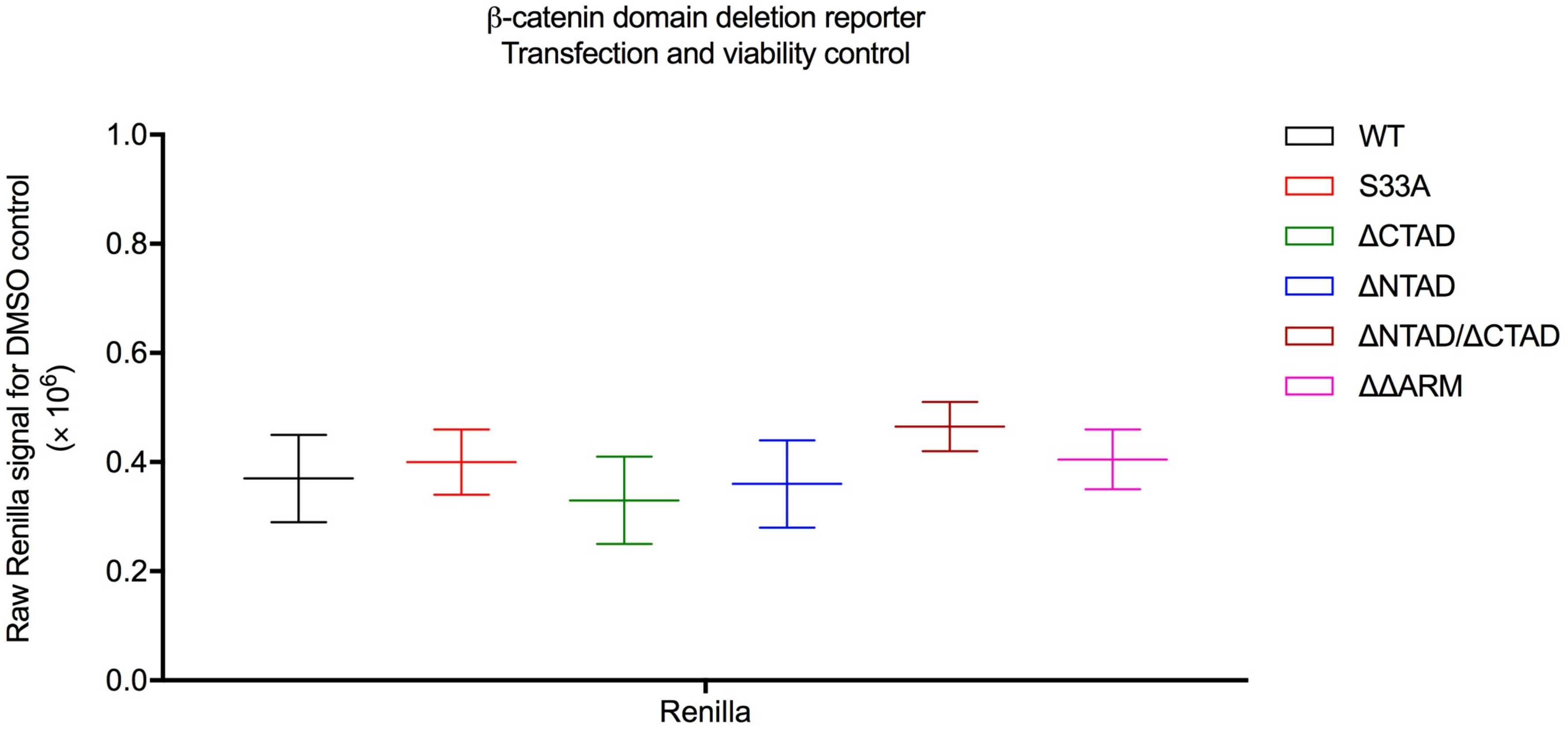

Supplementary Figure 7

C2: ΔNTAD interaction

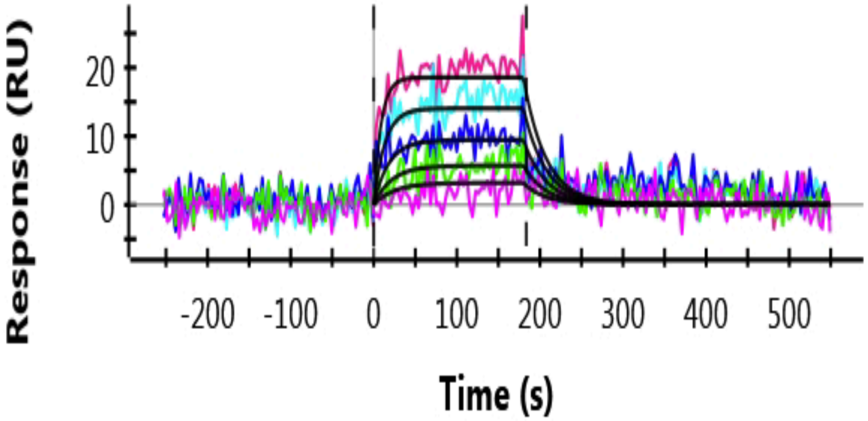

K_{on} = 3.8 × 10^{5} M/s; K_{off} = 4.2 × 10^{-3} 1/s; K_d = 1.1 × 10^{-8} M

C2: ΔCTAD interaction

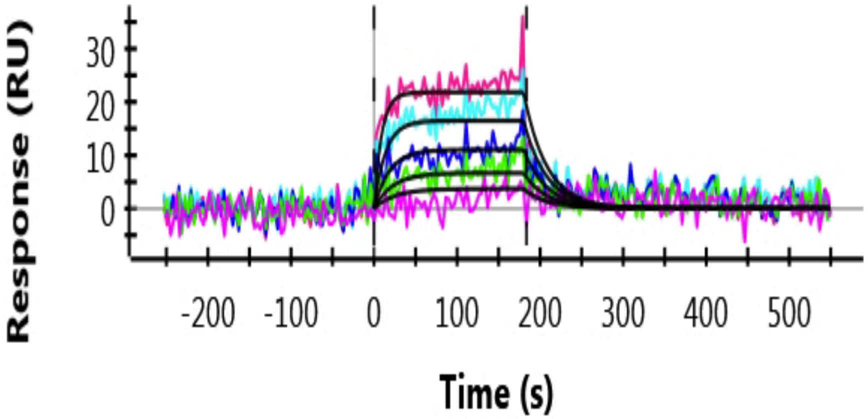

K_{on} = 3.2 × 10^{5} M/s; K_{off} = 3.9 × 10^{-3} 1/s; K_d = 1.21 × 10^{-8} M

C2: ΔNTAD / ΔCTAD interaction

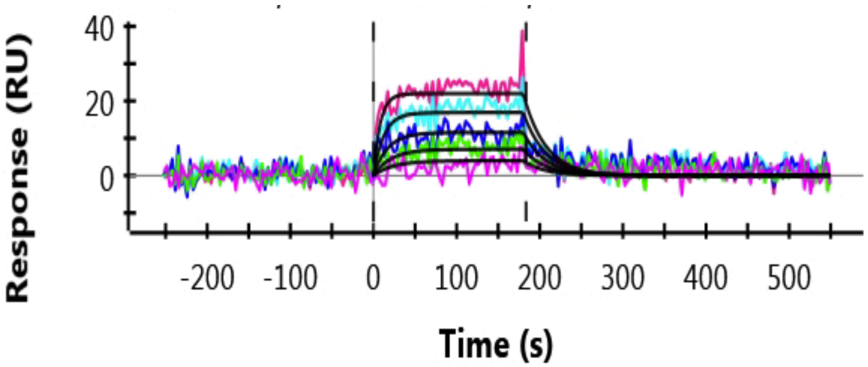

K_{on} = 3.1 × 10^{5} M/s; K_{off} = 4.5 × 10^{-3} 1/s; K_d = 1.45 × 10^{-8} M

C2: ΔARM interaction

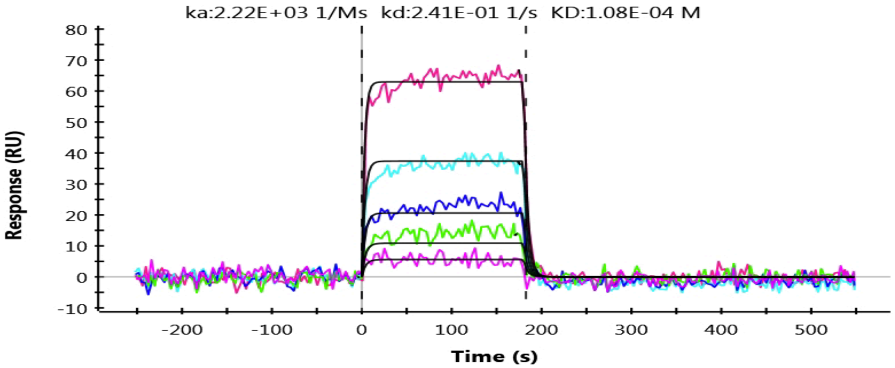

K_{on} = 2.2 × 10^{3} M/s; K_{off} = 2.4 × 10^{-1} 1/s; K_d = 1.08 × 10^{-4} M

Supplementary Figure 8

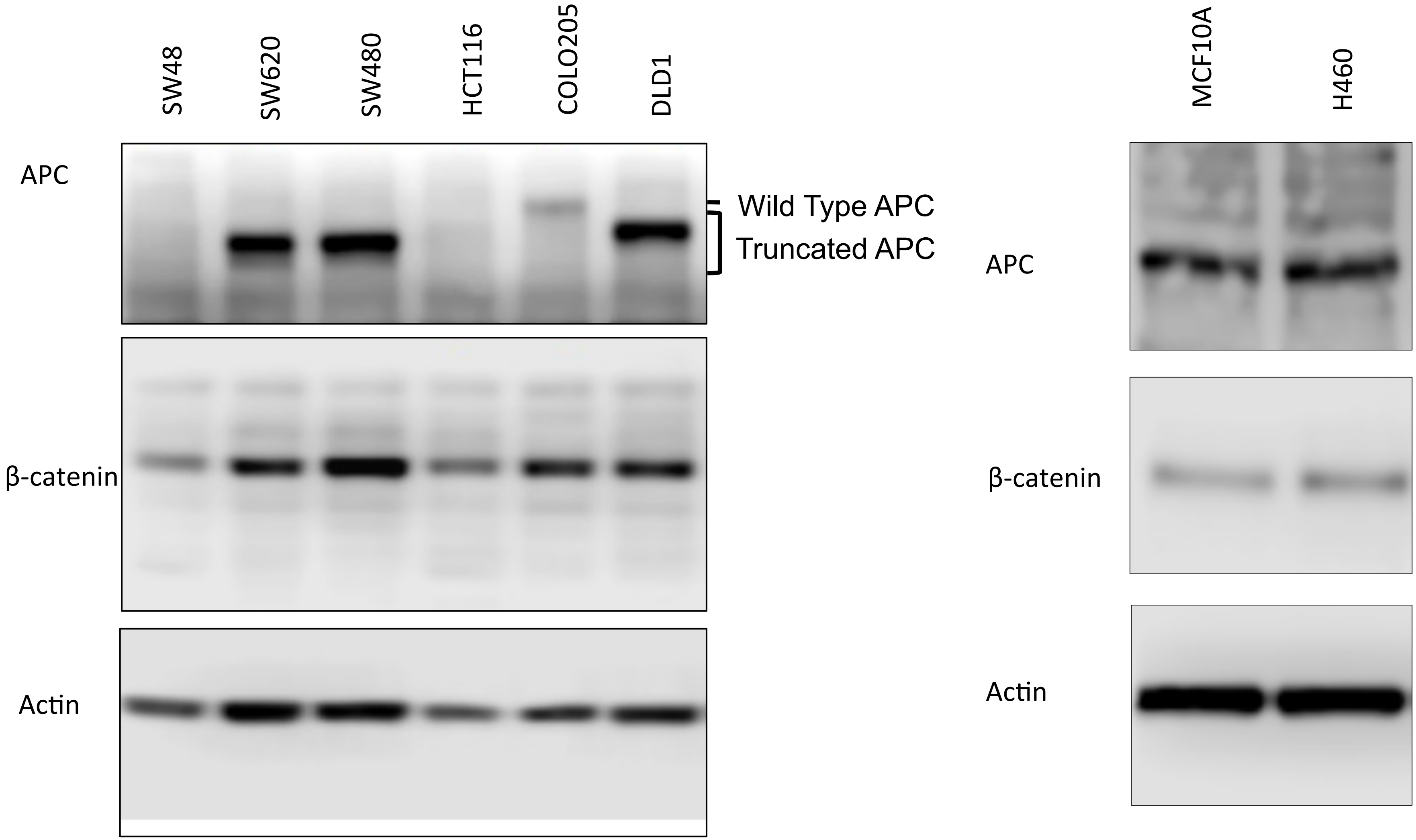

Supplementary Figure 9

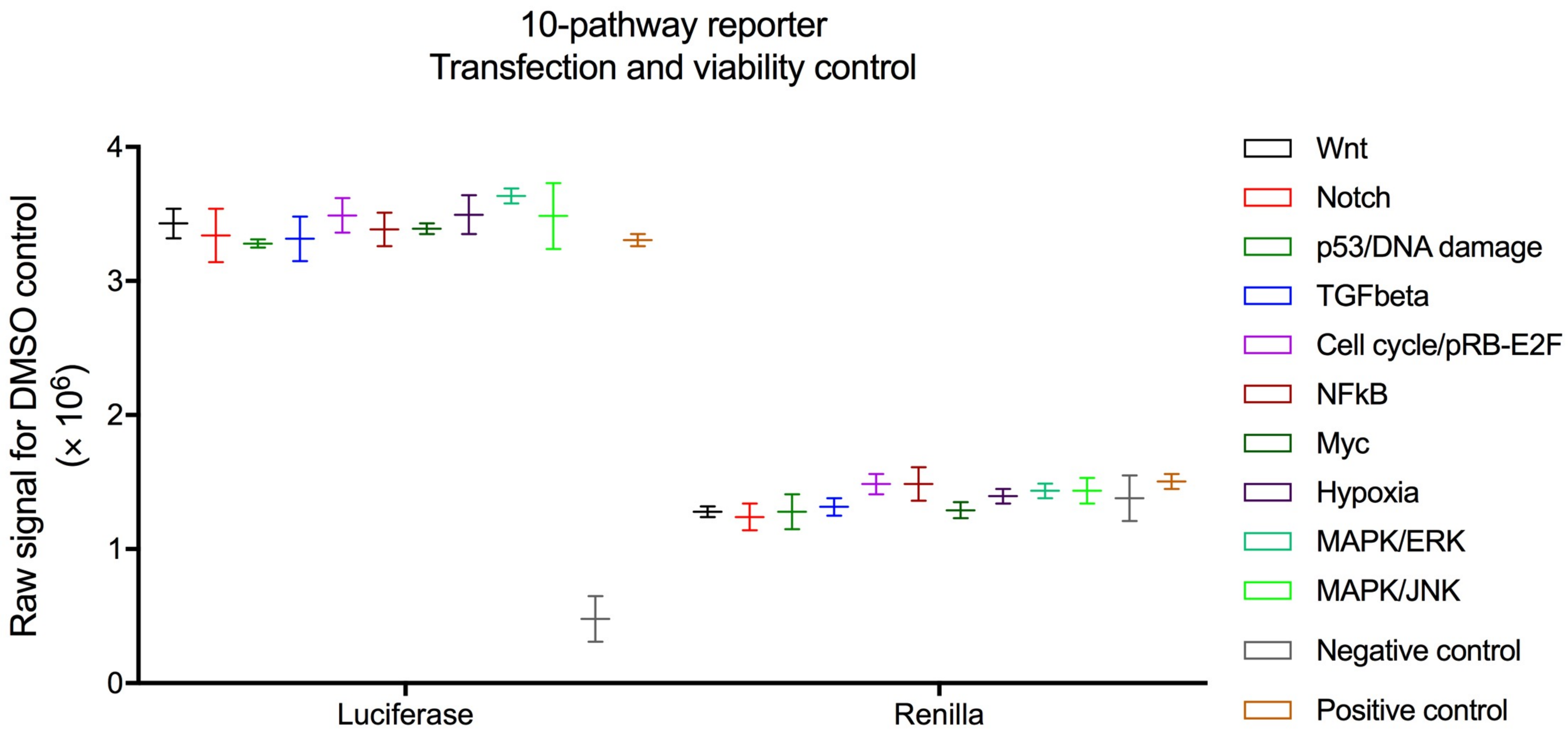

Supplementary Figure 10

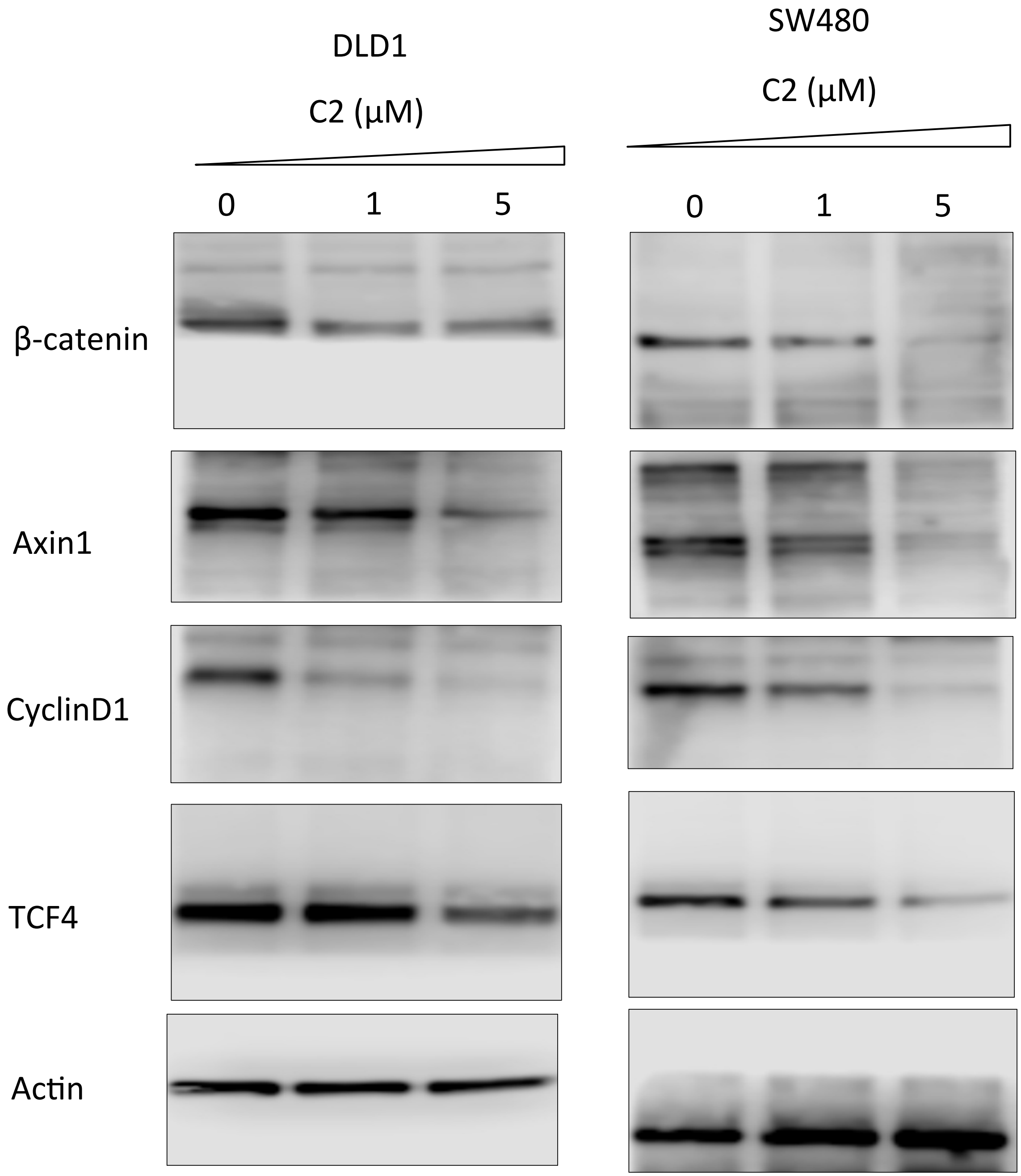

Supplementary Figure 11

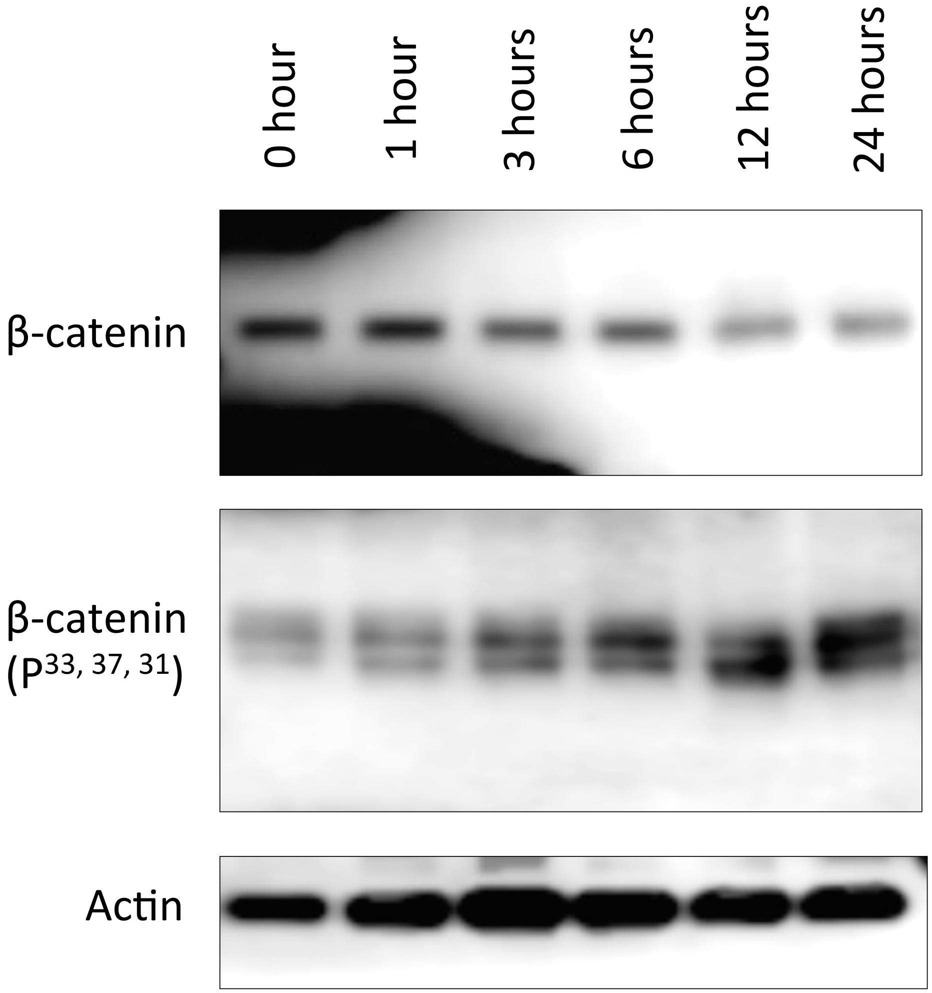

Supplementary Figure 12

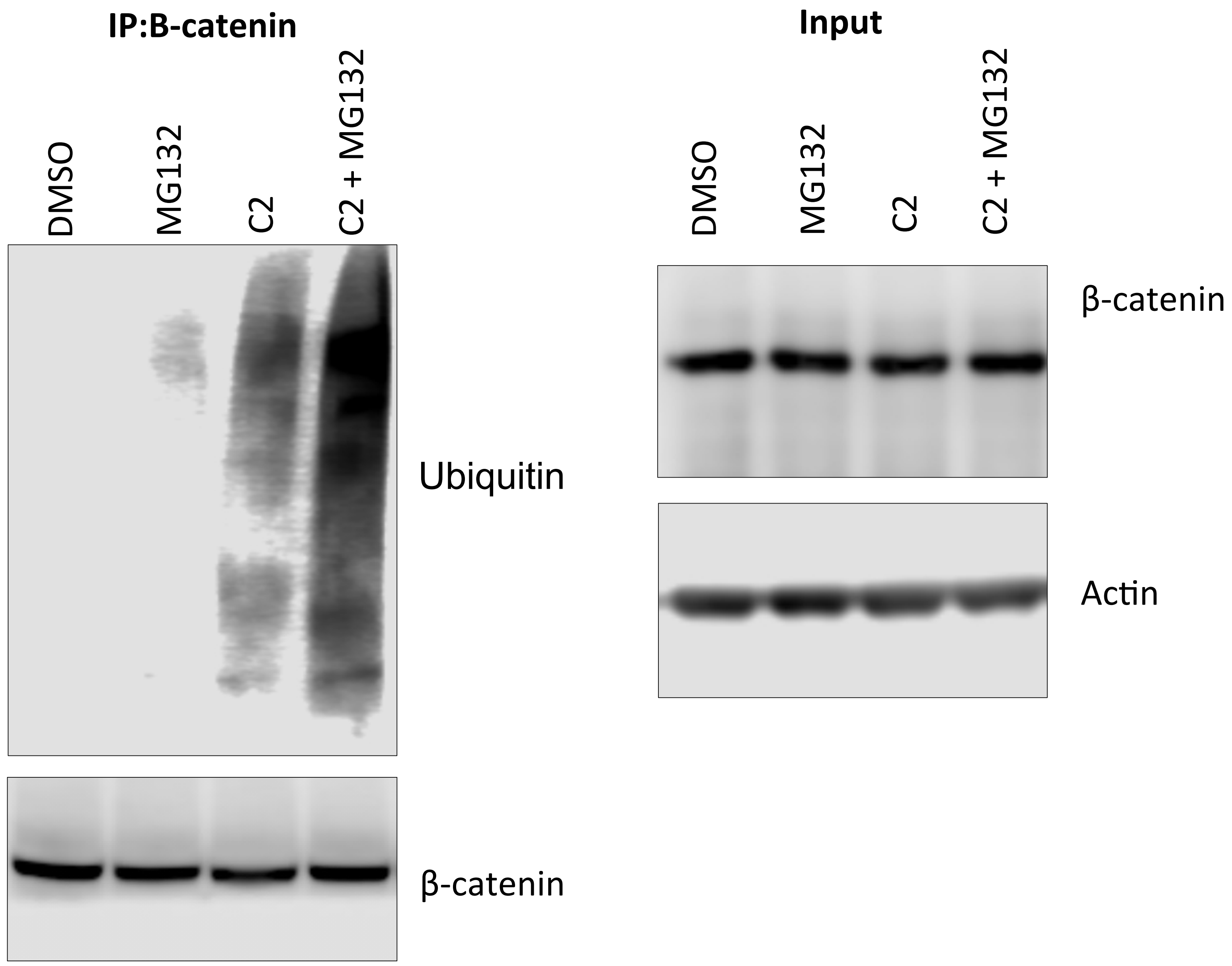

Supplementary Figure 14

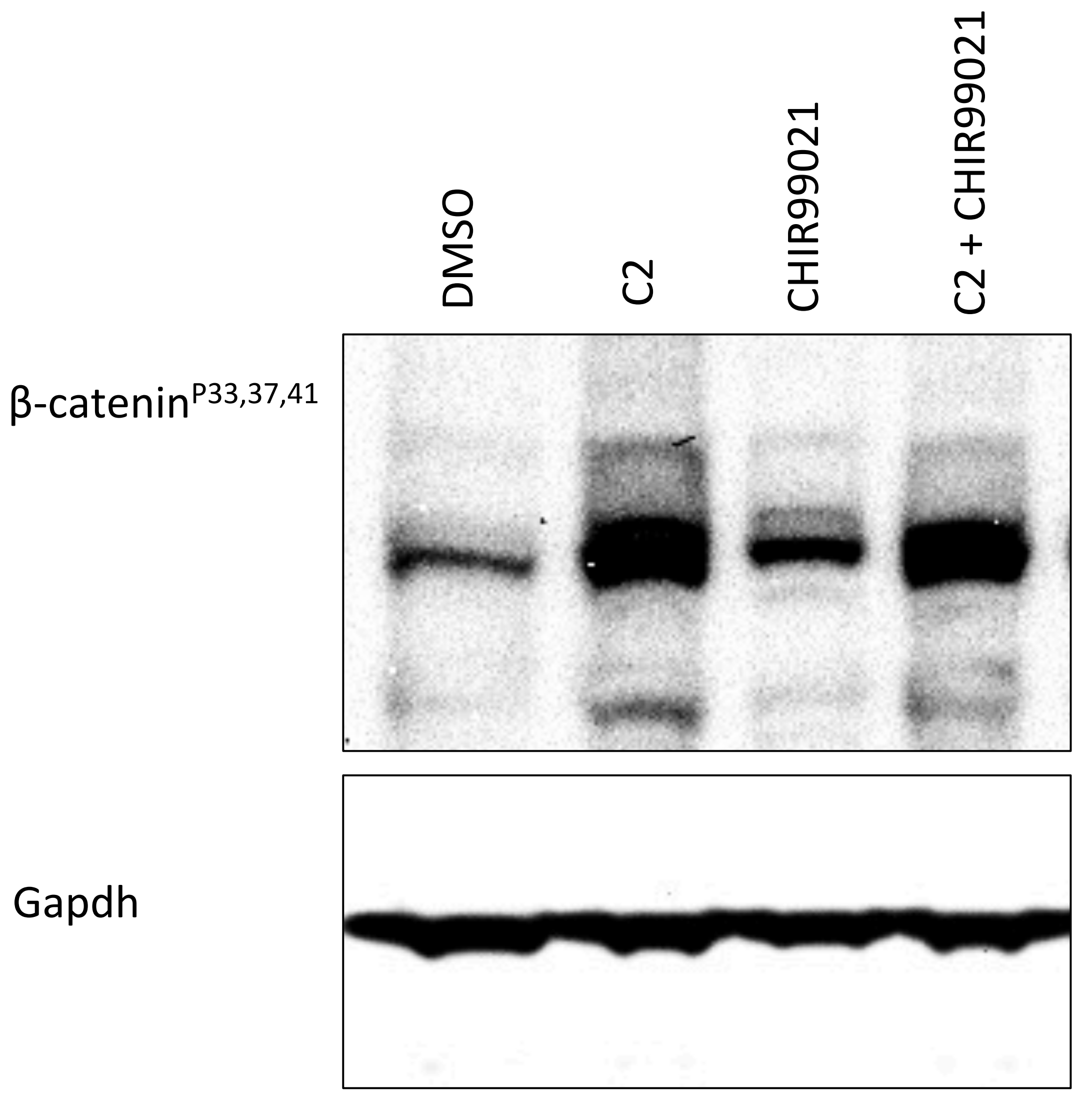

Supplementary Figure 13

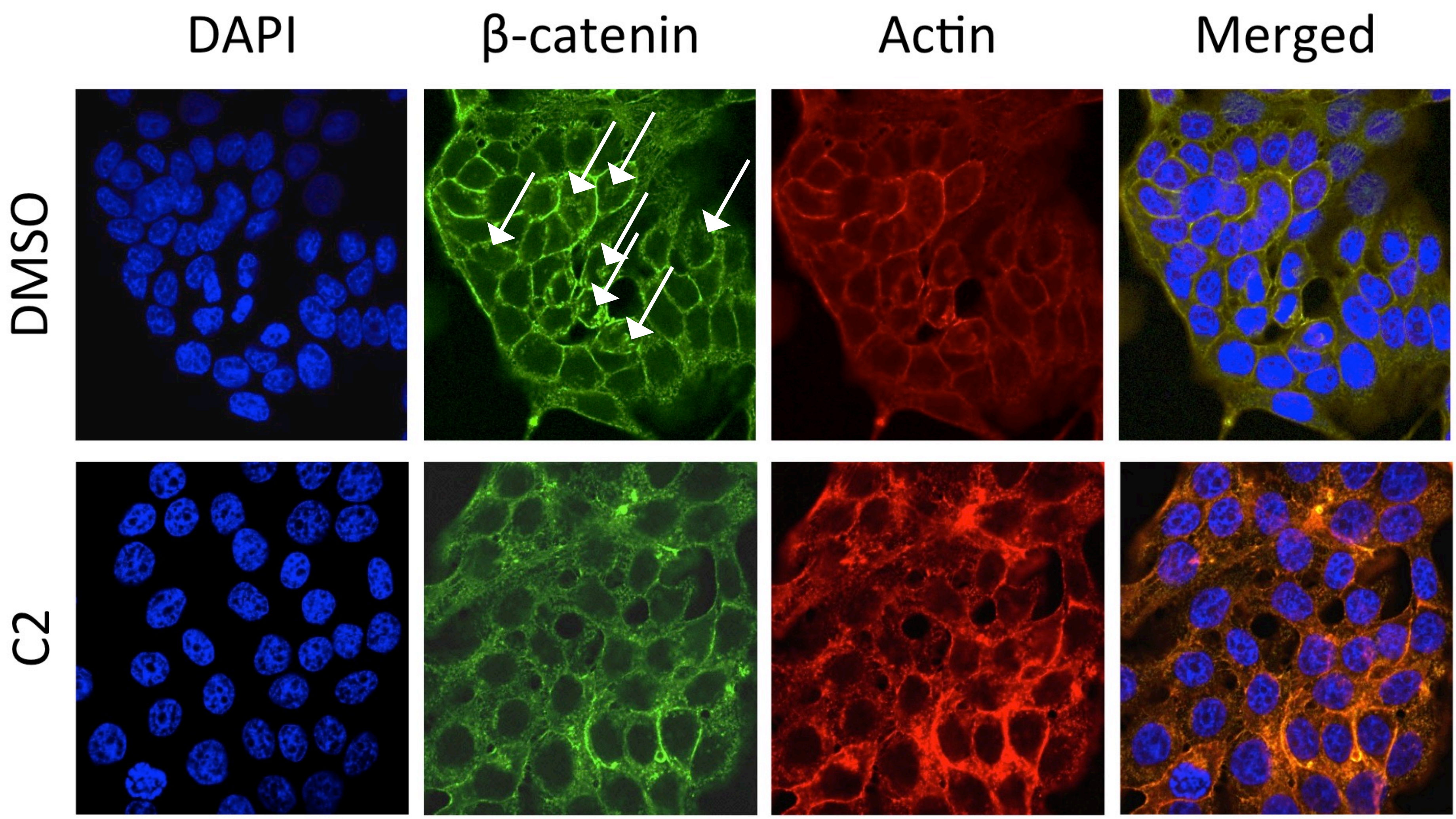

Supplementary Figure 15

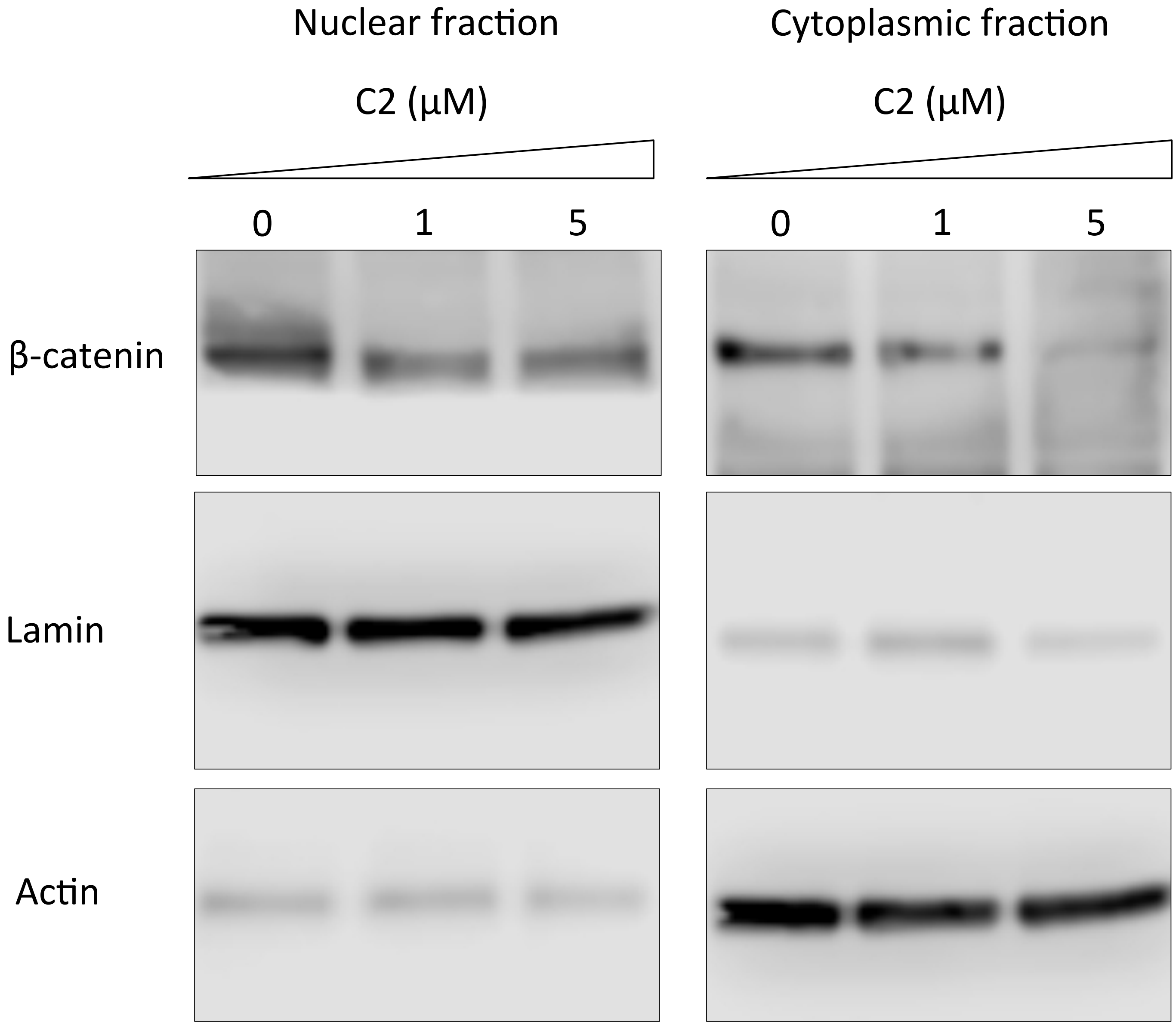

Supplementary Figure 16

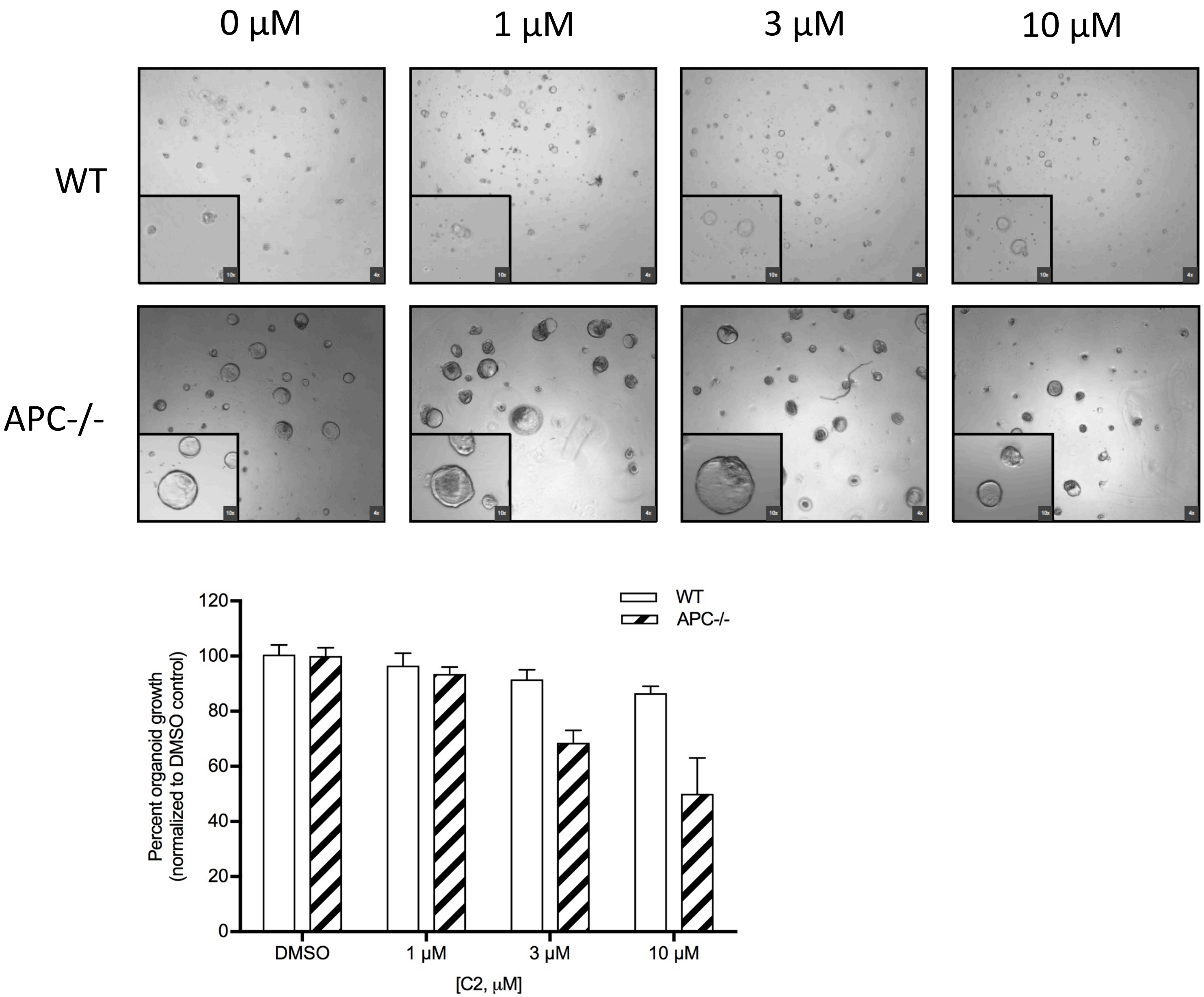

Supplementary Table 1

Compound Name	TopFlash Reporter Effect	NSC number
C1	Activator	331942
C2	Inhibitor	211416
C3	Activator	682520
C4	Activator	378705
C5	Activator	134142
C6	Activator	51478
C7	Activator	95619
C8	Activator	723449
C9	Activator	44585
C10	Activator	724299
C11	Inhibitor	736075
C12	Activator	50261
C13	Activator	699421
C14	Activator	724300
C15	Activator	663614
C16	Inhibitor	44582
